# WTAP participates in the DNA damage response via an m^6^A-FOXM1-dependent manner in hepatocellular carcinoma

**DOI:** 10.1038/s41420-025-02639-x

**Published:** 2025-08-22

**Authors:** Nan Huang, Zhixuan Bian, Chang Xu, Yue Zhang, Li Liu, Zhongqi Cui, Shasha Zhao, Qiangyuan Fan, Shaobo Xue, Yan Chen, Qiuhui Pan, Fenyong Sun

**Affiliations:** 1 Department of Clinical Laboratory Medicine, Shanghai Tenth People’s Hospital of Tongji University, Shanghai, 200072 China; 2 Department of Laboratory Medicine, Shanghai Children’s Medical Center, Shanghai Jiao Tong University School of Medicine, Shanghai, 200127 China; 3 Shanghai Key Laboratory of Clinical Molecular Diagnostics for Pediatrics, Shanghai, 200127 China; 4 Department of Central Laboratory, Clinical Medicine Scientific and Technical Innovation Park, Shanghai Tenth People’s Hospital, Shanghai, 200072 China

**Keywords:** Cancer epidemiology, DNA damage response

## Abstract

N6-Methyladenosine (m^6^A) is one of the most abundant RNA modifications that occur in eukaryotes. The relationship between m^6^A methylation and DNA damage repair (DDR) is still unclear. As an important chaperone protein of m^6^A methyltransferase, the role of Wilm’s tumor–associated protein (WTAP) in DDR has not been studied in detail. We identified that WTAP was markedly upregulated in HCC cells with DNA damage induction. Further investigations revealed that WTAP was engaged in DDR and WTAP knockdown resulted in a significant decrease of the stability of the DDR-related transcription factor Forkhead Box M1 (FOXM1) mRNA, eventually preventing DDR. WTAP deficiency inhibited cell growth both in vivo and in vitro. Moreover, WTAP silencing sensitized HCC cells to cisplatin, a well-known DNA damage agent. Altogether, our findings demonstrate that WTAP is the dominant m^6^A -related modulator upon DNA damage and functions by stabilizing FOXM1. In addition, WTAP deficiency saliently sensitized HCC cells to cisplatin both in vitro and in vivo.

## Introduction

Hepatocellular carcinoma (HCC) is one of the leading causes of cancer-associated deaths worldwide [1]. Although the incidence of HCC has gradually decreased in recent years, the mortality rate remains high due to a lack of appropriate treatments [2]. Liver transplantation and hepatectomy are a suitable treatment option for <20% of patients with HCC, and conventional radiation therapy remains the main method of ablative non-invasive treatment. However, the benefit of radiotherapy is limited due to innate or induced radiation resistance of HCC cells [3, 4].

Radiation induces double-strand breaks (DSB) in DNA, the most severe type of DNA damage, which can activate DNA damage repair (DDR) signaling pathways [4–6]. There are two main pathways for DSB repair: homologous recombination (HR) and non-homologous end joining (NHEJ). The elucidation of the molecular pathways involved in DNA damage signal transduction and repair may allow the identification and targeting of specific signaling molecules in the DDR pathway [7]. Moreover, previous studies [8–10] suggested that inhibiting the tumor cell response to DNA damage can compete with radiotherapy or chemotherapy resistance, and may serve as a promising therapeutic method.

N6-methyladenosine (m^6^A) methylation occurs at the transcriptional level in various RNA molecules. The modifications occur mostly in the stop codon and 3’UTR (untranslated region) region, therefore affecting RNA transcription, processing, translation, degradation, and other functions [11]. M^6^A modification is a dynamic and reversible process involving a series of enzymes, including methyltransferases (writers), demethyltransferases (erasers) and recognition proteins (readers) [12]. Abnormal expression or function of these m^6^A-related molecules causes a myriad of changes in downstream RNAs and may result in tumorigenesis. Two m^6^A demethylases have been identified so far, namely FTO (fat mass and obesity-associated protein) and ALKBH5 (α-ketoglutarate-dependent dioxygenase alkB homolog 5) [12]. The modification of m^6^A is primarily mediated by the METTL3 (methyltransferase like-3)-METTL14 (methyltransferase like-14)-WTAP (Wilm’s tumor-associated protein) complex. WTAP lacks catalytic activity, and functions as an adapter protein in the complex, exerting a crucial role in RNA metabolism [13]. A recent study suggested that WTAP was significantly upregulated in HCC, and that WTAP-mediated m^6^A modification played a salient role in HCC tumorigenesis [14]. However, the specific mechanisms of how m^6^A -related proteins participate in the DDR remain unknown.

Forkhead Box M1 (FOXM1) belongs to the forkhead box superfamily of transcription factors [15]. FOXM1 regulates the expression of DDR-related genes and contributes to radiotherapy and chemotherapy resistance [16, 17]. A recent study reported that ALKBH5 could demethylate FOXM1 nascent transcripts, thus increasing FOXM1 expression so as to promote tumorigenesis and development of glioblastoma [18]. Nevertheless, the upstream mechanism of FOXM1 regulation in HCC has not been fully elucidated.

It has been recently reported that the METTL3-m^6^A-YTHDC1 axis regulates the accumulation of DNA-RNA hybrids at DNA damage sites, and subsequently recruits key factors for HR-mediated repair [19]. However, there is currently no direct evidence showing that WTAP and m^6^A serve a role in the repair of DNA damage. Herein, we found that the m^6^A modification level was significantly upregulated when DNA damage were induced and WTAP was the most prominent gene among all the m^6^A writers in HCC. Further investigations revealed that the high expression of WTAP in HCC could facilitate DDR efficiency by enhancing the m^6^A level and stability of FOXM1 mRNA which was engaged in DDR by promoting the transcription of DNA damage related proteins. This study can provide new ideas and therapeutic targets for the prevention of tumor cell resistance to radiotherapy and chemotherapy in HCC.

## Results

### M^6^A-related enzymes are associated with DDR

**Fig. 1:**
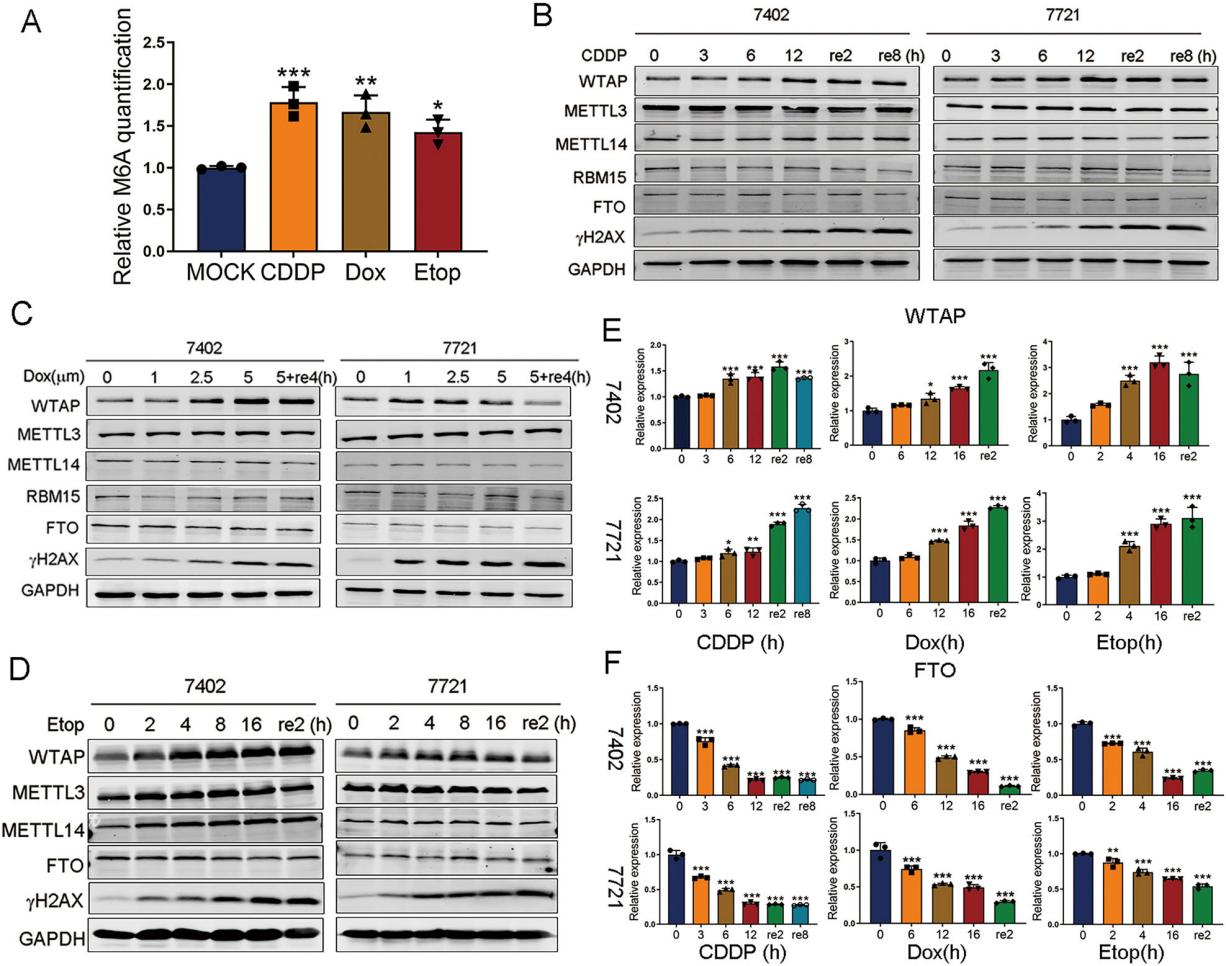
Association between m^6^A-related enzymes and DNA damage response. **A** Elevation of m^6^A methylation level of BEL-7402 cells incubated with 3 different DNA damage induced drugs for 12 h (10 μg/ml CDDP, 5 μM Dox, and 50 μM Etop) determined by m^6^A enzyme-linked immunosorbent assay. **B**−**D** The protein expression of WTAP, METTL3, METTL14, FTO, γH2AX or RBM15 in HCC cells (BEL-7402 and SMMC-7721) with the treatment of CDDP (10 μg/ml for 0, 3, 6, 12 h followed by 4 h or 8 h recovery) (**B**), Dox (0, 1, 2.5, 5 μM for 12 h followed by 4 h recovery), or Etop (50 μM for 0, 2, 4, 8, 16 h followed by 2 h recovery). **E** The mRNA expression of WTAP in HCC cells (BEL-7402 and SMMC-7721) with the treatment of CDDP (10 μg/ml for 0, 3, 6, 12 h followed by 4 h or 8 h recovery), Dox (1 μM for 0, 2, 4, 16 h followed by 2 h recovery), or Etop (50 μM for 0, 2, 4, 16 h followed by 2 h recovery). **F** The mRNA expression of FTO in HCC cells (BEL-7402 and SMMC-7721) with the treatment of CDDP (10 μg/ml for 0, 3, 6, 12 h followed by 4 h or 8 h recovery), Dox (1 μM for 0, 2, 4, 16 h followed by 2 h recovery), or Etop (50 μM for 0, 2, 4, 16 h followed by 2 h recovery). All the results were obtained from at least three independent experiments. *p < 0.05, **p < 0.01, ***p < 0.001 compared with MOCK, 0 h or 0 μM. re2 (h), 2 h recovery; re8 (h), 8 h recovery; 5 + re4 (h), 5 μM followed by 4 h recovery.

To investigate the relationship between m^6^A methylation and DDR, HCC cells were treated with three DNA damage inducing drugs. Results from the colorimetric assay and dot blot assay suggested that the global m^6^A level in the cells was increased following induction of DNA damage (Fig. 1A, Fig. S1A, B). We further detected the protein levels of m^6^A-related enzymes in HCC cells treated with CDDP, DOX or ETOP at the indicated time points. Results from western blot showed that the protein level of WTAP increased significantly in response to DDR induced by the three drugs, while the protein levels of METTL3, METTL14, FTO or RBM15 were not affected (Fig. 1B–D). We also evaluated the mRNA levels of these genes and found a striking increase in WTAP mRNA expression after exposure to CDDP and ETOP (Fig. 1E), as well as a slight elevation in METTL3 and METTL14 mRNA levels (Fig. S1C–F). Downregulation of FTO mRNA levels was also observed (Fig. 1F). Collectively, the results suggested that the global m^6^A modification level was increased when DNA damage were induced, and that WTAP may be the most important m^6^A modulator in HCC.

### WTAP is involved in DDR in HCC cells

**Fig. 2:**
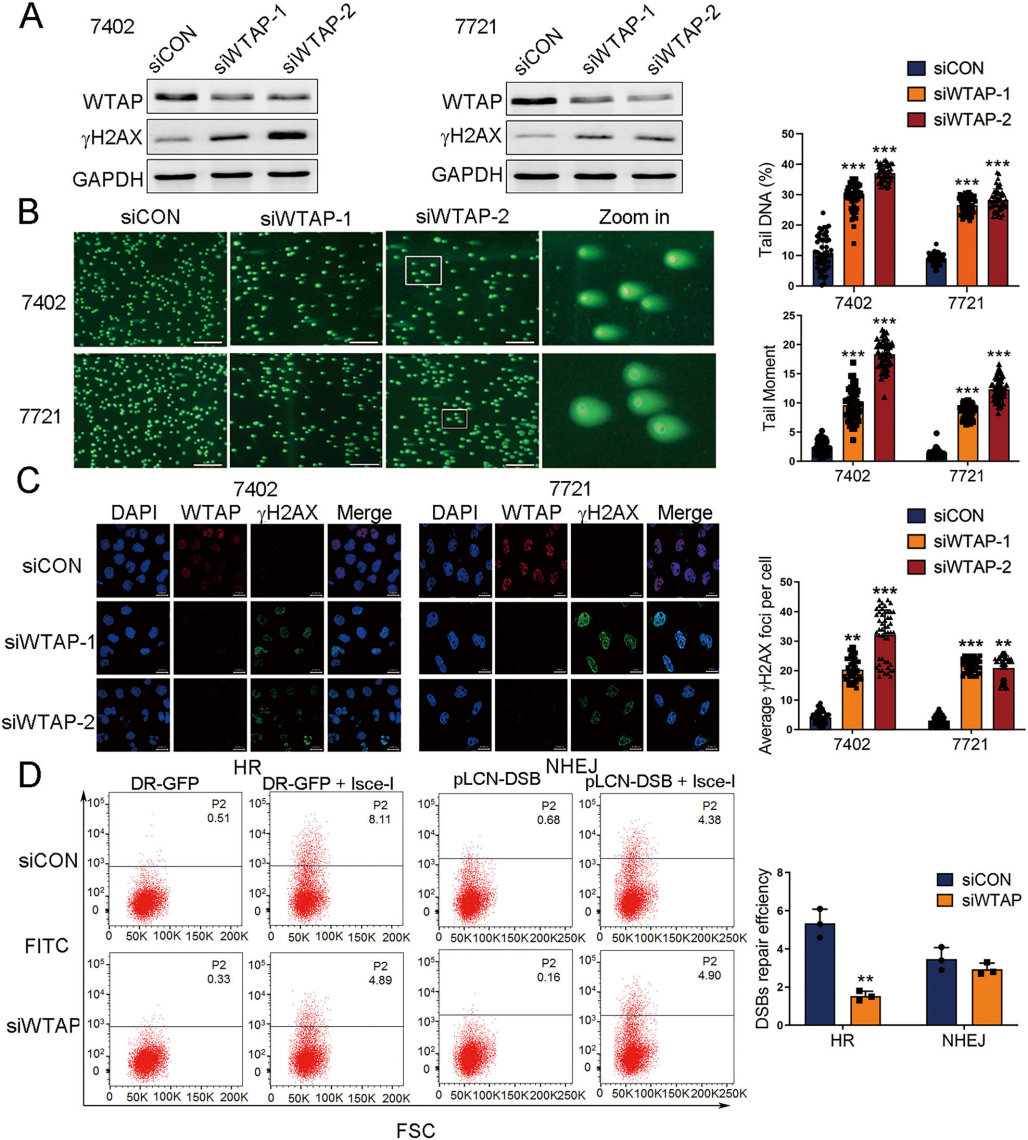
The engagement of WTAP in DDR. **A** The efficiency of WTAP knockdown and protein level of γH2AX in two HCC cell lines detected by western blot. **B** Representative images and quantification of tail DNA observed in siCON or siWTAP cells by comet assay (*n* = 50, Mann-Whitney test). Scale bar, 500 μm. **C** Immunofluorescence analysis of the formation of γH2AX foci in siCON or siWTAP HCC cells (*n* = 50, Mann-Whitney test). Scale bar, 20 μm. **D** HR and NHEJ efficiency of siCON or siWTAP HCC cells detected by flow cytometry. All results were obtained from at least three independent experiments. **p < 0.01, ***p < 0.001 compared with siCON.

To clarify the role of WTAP in DDR following the induction of DNA damage, we first detected levels of WTAP in a series of HCC cell lines and the normal liver cell line (HL-7702). Our results showed higher expression of WTAP in BEL-7402 and SMMC-7721 cells compared to other HCC cell lines and the normal liver cell line (Fig. S2A). Therefore, we further knocked down the expression of WTAP in BEL-7402 and SMMC-7721 cells with siRNAs against WTAP (Fig. 2A), and investigated the changes in DDR efficiency in WTAP-silenced HCC cells (BEL-7402 and SMMC-7721). Results from western blot revealed that the protein expression of the DNA damage-related gene γH2AX was significantly increased in WTAP-silenced cells (Fig. 2A), suggesting that inhibition of WTAP expression hindered DDR. Furthermore, an increase of tail DNA in WTAP-silenced cells was observed in the comet assay (Fig. 2B). Immunofluorescence confirmed a significant elevation of γH2AX foci with the interference of WTAP in HCC cells (Fig. 2C). Moreover, a reporter analysis was performed to determine the type of DDR that WTAP participated in. The results revealed that WTAP deficiency significantly reduced the efficiency of HR but not that of NHEJ (Fig. 2D).

### Screening of differentially-methylated genes in WTAP-knockdown HCC cells upon DNA damage

**Fig. 3:**
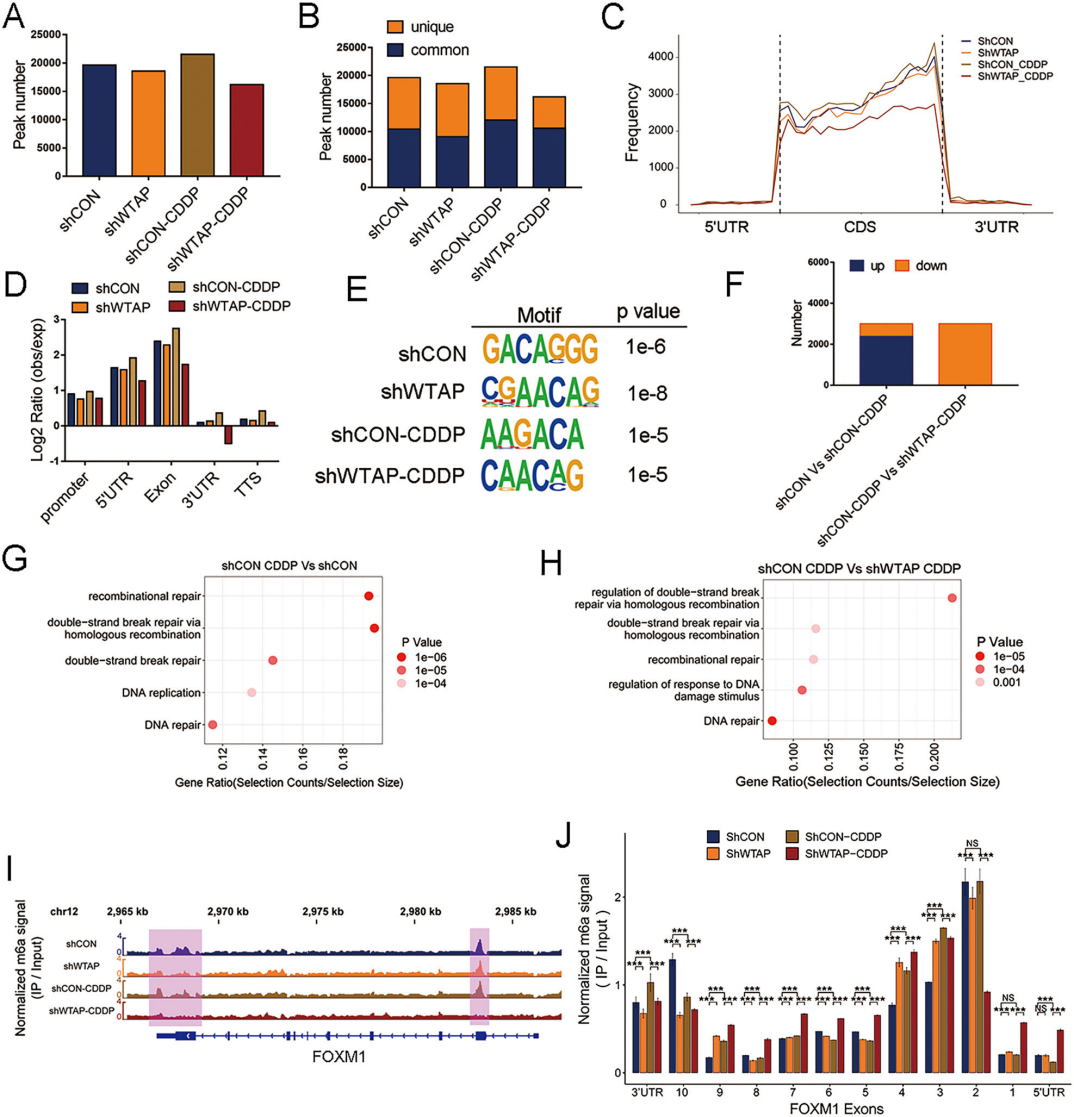
Screening of differentially methylated genes after WTAP knockdown and simultaneously CDDP-induced DNA damage. **A** Number of m^6^A peaks identified in MeRIP-seq in shCON, shWTAP, shCON+CDDP and shWTAP+CDDP cells. **B** Composition of m^6^A peaks identified in MeRIP-seq. Common: m^6^A genes contain at least 1 common m^6^A peak, unique: m^6^A genes contain no common m^6^A peaks. **C** Distribution of m^6^A peaks identified in four groups across transcripts with different regions which are separated into 5’ untranslated region (5’UTR), coding region (CDS) and 3’ untranslated region (3’UTR). **D** Quantification of m^6^A peaks identified in different regxp: expected peaks. **E** Top consensus motif identified in four groups respectively analyzed by HOMER analysis with MeRIP-seq data. **F** Histograms represented number of differentially methylated genes in different groups (shCON *vs* shCON-CDDP; shCON-CDDP *vs* shWTAP-CDDP). **G** A cluster profiler of enriched gene ontology processes of differentially methylated genes in CDDP-treated cells compared with the control. **H** A cluster profiler of enriched gene ontology processes of differentially methylated genes in CDDP-treated and WTAP-deficient cells compared with CDDP-treated control cells. **I** Distribution of m^6^A peaks across FOXM1 mRNA transcript analyzed by Integrative Genomics Viewer (IGV). The most obvious differences in 3’UTR and the second exon were highlighted in purple. **J** The m^6^A signal identified in 10 exons of FOXM1. ***p < 0.001 compared with shCON, or shCON-CDDP. NS, no sense.

We first performed dot blot assay to measure the overall level of m6A in HCC cells with stable knockdown of WTAP or the corresponding control, and observed levels of m6A decreased with the knockdown of WTAP in both BEL-7402 and SMMC-7721 cells (Fig. S2B). To obtain an mRNA transcriptome-wide m^6^A profile associated with WTAP and DNA damage, we then performed MeRIP-seq analysis using WTAP-knockdown BEL-7402 cells upon CDDP-induced DNA damage. MeRIP-seq identified 18565 m^6^A peaks of 6544 genes in WTAP-knockdown cells, which was slightly less than the control (19634 m^6^A peaks of 7229 genes). As expected, the highest number of peaks (21539 m^6^A peaks of 7439 genes) was identified in cells treated with CDDP, and the smallest number of peaks (16202 m^6^A peaks of 5828 genes) was seen in WTAP-knockdown cells exposed to CDDP (Fig. 3A, B), which was consistent with the result shown in Fig. 1A. These results indicated that m^6^A modification was closely related to DNA damage, and that WTAP might play a synergistic role in the process. The distribution of m^6^A peaks was abundant in the protein coding sequence, and highly enriched around stop codons and 5’ untranslated regions (5’UTR) (Fig. 3C, D), which was consistent with the conventional m^6^A distributions within mRNA [20]. The enriched m^6^A sites identified was coincident with the conserved m^6^A motif RRACH (R = A or G; H = A, U, or C) (Fig. 3E) [20]. We identified a variety of m^6^A-modified genes that were significantly altered upon CDDP treatment or WTAP deficiency (Fig. 3F, Supplementary Table S1). GO analysis of the differentially- m^6^A-modified transcripts identified in the control and CDDP-treated cells indicated that these genes were enriched in DDR (Fig. 3G). Notably, differential m^6^A genes identified in WTAP-knockdown and CDDP-treated cells were also enriched in the regulation of DSB, especially in HR (Fig. 3H), which was consistent with WTAP serving a role in HR as shown in Fig. 2. Surprisingly, we identified that the m^6^A modification of FOXM1, a transcription factor closely related to DDR [21–23], was increased after CDDP treatment but decreased after WTAP-knockdown and CDDP treatment (Supplementary Table S1). More intriguingly, we observed that the 3’UTR region of FOXM1 had a high abundance of m^6^A peaks, which decreased following WTAP-knockdown and CDDP treatment (Fig. 3I). The abundance of m^6^A modifications in different exons of FOXM1 was shown in Fig. 3J. In brief, m^6^A modifications mediated by WTAP participate in the induction of DNA damage, and FOXM1 may be the downstream target of WTAP, and therefore serves an important role in DDR.

### WTAP regulates FOXM1 mRNA stability in an m^6^A-dependent manner

**Fig. 4:**
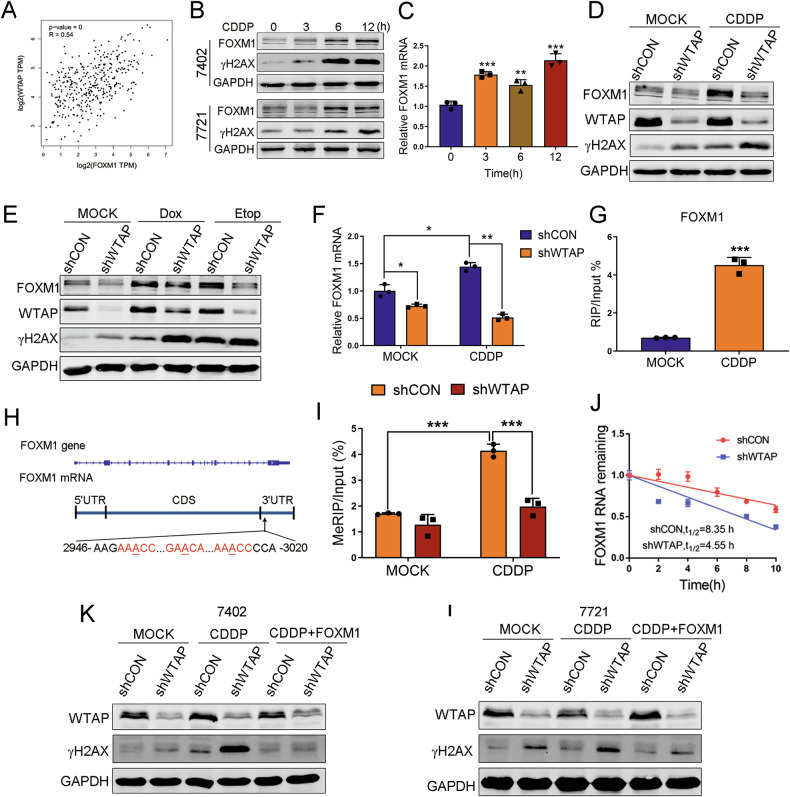
WTAP affects FOXM1 mRNA stability *via* m^6^A modification. **A** Correlation analysis from GEPIA of WTAP and FOXM1 mRNA expression in HCC. **B**, **C** The protein expression and relative mRNA expression of FOXM1 in two HCC cell lines treated with 10 μg/ml CDDP at indicated time points. **D**, **E** The protein expression of FOXM1 in WTAP-deficient HCC cells treated with 3 different DNA damage induced drugs for 12 h (10 μg/ml CDDP, 5 μM DOX, and 50 μM ETOP). **F** The relative mRNA expression of FOXM1 in WTAP-knockdown and CDDP-induced cells determined by RT-qPCR. **G** Relative levels of FOXM1 mRNA immunoprecipitated with anti-WTAP. The mRNA level was normalized against expression in the mock group. **H** The schematic diagram showed the site of the FOXM1 primers used in MeRIP-qPCR. **I** Levels of FOXM1 mRNA immunoprecipitated with anti-m^6^A in four indicated groups. **J** The half-life and stability of FOXM1 mRNA in WTAP-deficient HCC cells compared with control cell. **K**, **L** Rescue assays showed the effect of FOXM1 overexpression on DDR in WTAP-knockdown and CDDP-treated HCC cells. All the results were obtained from at least three independent experiments. *p < 0.05, **p < 0.01, ***p < 0.001 compared with 0 h, shCON, or MOCK.

To further investigate whether WTAP was involved in DDR by modulating FOXM1 in an m^6^A-mediated manner, we used the Gene Expression Profiling Interactive Analysis (GEPIA, http://gepia.cancer-pku.cn/index.html) database to identify associations between WTAP and FOXM1 in HCC tissues. As shown in the scatter plot in Fig. 4A, a significant positive correlation was observed between the two genes. Following exposure to CDDP and the induction of DNA damage, the protein level of FOXM1 increased as the DNA damage became more severe (Fig. 4B), most likely due to the increase in FOXM1 mRNA expression (Fig. 4C). Moreover, FOXM1 was downregulated in WTAP-knockdown HCC cells, which was more pronounced when DNA damage was induced following exposure to CDDP, DOX or ETOP (Fig. 4D, E), similar to the RT-qPCR results presented in Fig. 4F. Since WTAP is one of the key writers involved in m^6^A modification, we speculated that WTAP might increase the mRNA and protein expression of FOXM1, especially in the presence of DNA damage. Thus, we performed RIP-qPCR to confirm the direct binding between the WTAP protein and FOXM1 mRNA. A strong interaction was observed following CDDP-induced DNA damage (Fig. 4G). As differential signals were detected in the 3’UTR of FOXM1 mRNA (Fig. 3I), we designed a specific primer in this region and performed MeRIP followed by RT-qPCR to evaluate FOXM1 expression in HCC cells with or without WTAP-knockdown in the absence or presence of CDDP treatment (Fig. 4H). The result verified that the reduction of WTAP led to a slight decrease of m^6^A enrichment in FOXM1, which was significantly decreased following exposure to CDDP (Fig. 4I). Moreover, the mRNA stability assay demonstrated that WTAP-knockdown destabilized levels of FOXM1 mRNA (Fig. 4J). Taken together, FOXM1 has been demonstrated to be the downstream target of WTAP and is regulated by WTAP in an m^6^A-dependent manner.

As no significant co-localization was observed between WTAP and γH2AX following exposure to CDDP (Fig. S3A), and we determined that FOXM1 was modulated by WTAP, we subsequently investigated whether WTAP regulated DDR by stabilizing FOXM1. Results from western blot showed that FOXM1-deficient cells exhibited decreased expression of the DDR-related genes NBS1 and RAD51, and increased expression of γH2AX (Fig. S3B). Results of immunofluorescence showed an increase of γH2AX foci with FOXM1 silencing, which further increased following exposure to CDDP (Fig. S3C). These results indicate that FOXM1 is involved in DDR, which is concordant with several previous studies [23, 24]. Furthermore, rescue assays were performed to verify that FOXM1 was the target of WTAP, and the results showed that WTAP knockdown followed by exposure to CDDP increased the expression of γH2AX, which could be partially blocked by FOXM1 overexpression (Fig. 4K, L). Altogether, our findings indicate that FOXM1 is a downstream target of WTAP and is involved in DDR in HCC. Additionally, the stability of FOXM1 mRNA is regulated by WTAP in an m^6^A-dependent manner.

### WTAP plays an oncogenic role in HCC

**Fig. 5:**
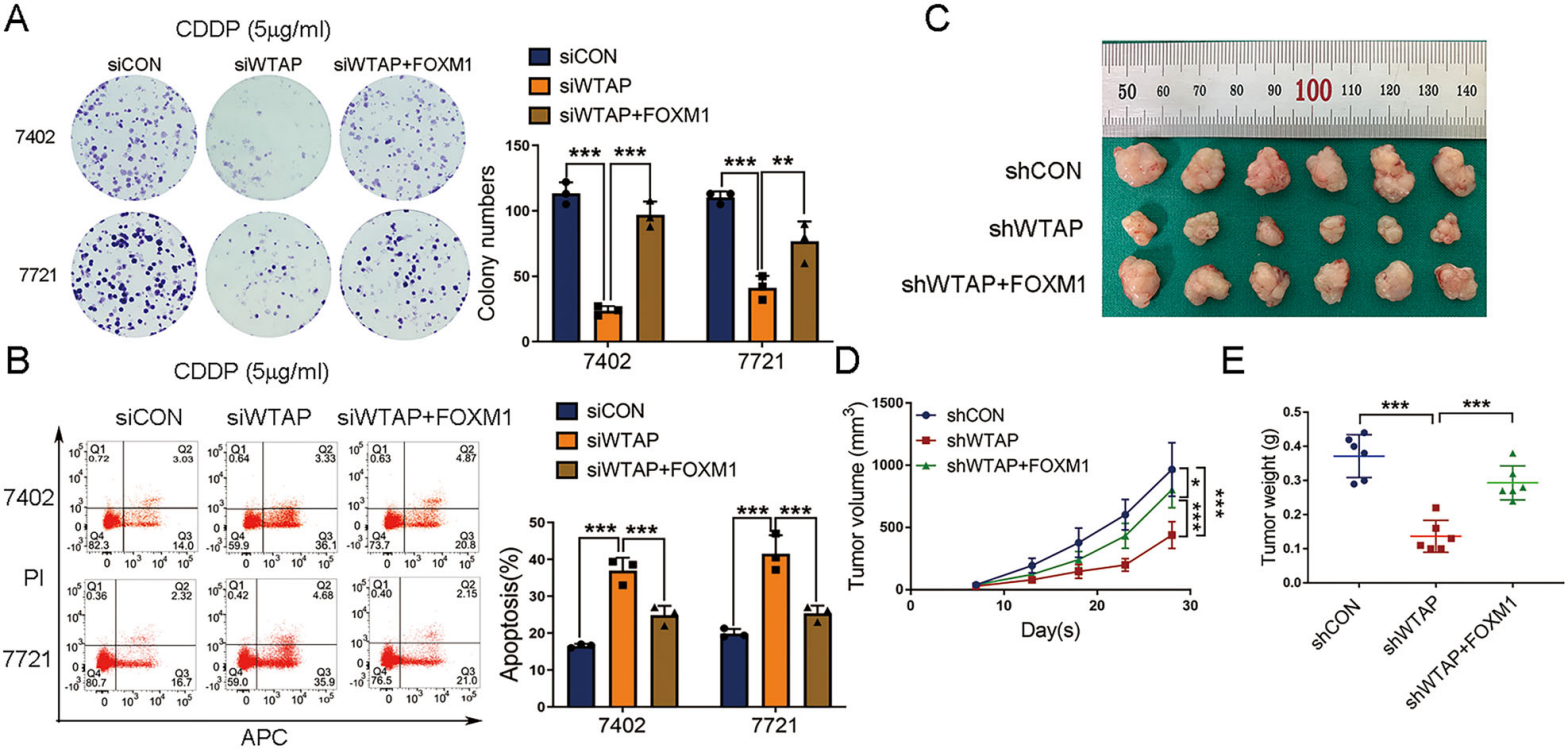
FOXM1 rescues the phenotype observed upon WTAP deficiency in vitro and in vivo. **A** Colony staining and (**B**) flow cytometry (A214-01 kit) to detect colony formation ability and cell apoptosis of HCC cells (BEL-7402 and SMMC-7721) cotransfected with siCON, siWTAP or siWTAP together with FOXM1 (siWTAP+FOXM1) followed by exposure to CDDP (5 μg/ml for 12 h). **C** Subcutaneous tumor xenograft model of BEL-7402 cells with stable WTAP knockdown (shWTAP), WTAP knockdown together with FOXM1 overexpression (shWTAP+FOXM1), or the vehicle control (shCON). **D**, **E** Tumor volume and weight were measured at indicated day points after cells were transplanted into mice. All the results were obtained from at least three independent experiments. *p < 0.05, **p < 0.01, ***p < 0.001 compared with siCON, siWTAP, shCON or shWTAP.

Analysis from the GEPIA database showed that WTAP was highly expressed in various tumors including HCC (Fig. S4A, B). In addition, Kaplan-Meier survival curves revealed that high WTAP expression was associated with decreased overall survival rates in patients with HCC (*p* = 6.5 × 10^−6^) (Fig. S4C). Subsequent analysis showed that WTAP expression was related to tumor grade and nodal metastasis status (Fig. S4D, E), suggesting that WTAP acts as an oncogene in HCC. We then established stable WTAP-knockdown cells using two independent shRNAs. The efficiency of knockdown was shown in Fig. S5A, B. Our results showed that knockdown of WTAP in both BEL-7402 and SMMC-7721 cells significantly inhibited cell proliferation and colony formation (Fig. S5C–E), and promoted cell apoptosis (Fig. S5F). We further examined the function of WTAP in vivo by establishing a xenograft tumor model in nude mice using WTAP-knockdown BEL-7402 cells or the negative control cells. A significant reduction in tumor volume and weight in the WTAP-knockdown group was observed compared with the control group (Fig. S5G–I). Collectively, these findings corroborate the oncogenic role of WTAP in HCC. To validate the involvement of FOXM in WTAP-medicated effects on HCC cells, we performed rescue assays. Our results showed that WTAP knockdown followed by exposure to CDDP induced inhibition of colony formation, and promotion of cell apoptosis in HCC cells, which was partially rescued by FOXM1 overexpression (Fig. 5A, B). Moreover, we subcutaneously injected BEL-7402 cells with WTAP knockdown (shWTAP), WTAP knockdown together with FOXM1 overexpression (shWTAP+FOXM1), or the vehicle control (shCON) into nude mice and determined the growth of tumor in vivo by monitoring tumor size weekly. Our results showed that overexpression of FOXM1 in subcutaneous xenograft tumor model rescued the inhibition of growth observed upon WTAP deficiency in vivo (Fig. 5C–E).

### WTAP knockdown increases the sensitivity of HCC cells to CDDP

**Fig. 6:**
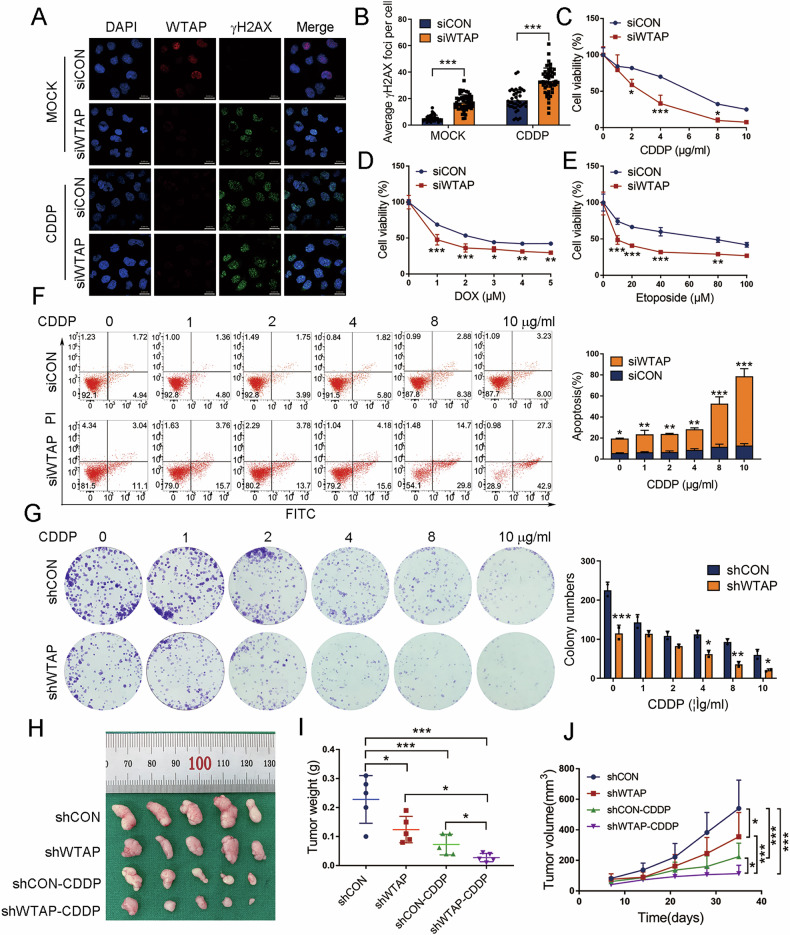
WTAP interference enhances the sensitivity of HCC cells to CDDP. **A**, **B** Immunofluorescence analysis and subsequent quantification of the formation of γH2AX foci in siCON and siWTAP cells incubated with or without 10 μg/ml CDDP (12 h followed by 2 h recovery) (*n* = 50, Mann-Whitney test). Scale bar, 20 μm. **C**–**E** CCK8 assays to detect cell viability in siCON and siWTAP cells treated with increasing doses of CDDP (0, 1, 2, 4, 8, 10 μg/ml), DOX (0, 1, 2, 3, 4, 5 μM), and ETOP (0, 10, 20, 40, 80, 100 μM) for 12 h. **F**, **G** Flow cytometry (BD 556547 kit) and colony staining to detect cell apoptosis and colony formation ability in siCON and siWTAP cells stimulated with CDDP at indicated concentration (0, 1, 2, 4, 8, 10 μg/ml) for 12 h. **H** Subcutaneous tumor xenograft model of SMMC-7721 cells with or without WTAP knockdown and with CDDP or saline solution treatment. **I**, **J** Tumor volume and weight were measured at indicated day points after cells were transplanted into mice. All the results were obtained from at least three independent experiments. *p < 0.05, **p < 0.01, ***p < 0.001 compared with siCON, shCON, shWTAP, or shCON-CDDP.

Given that WTAP is closely related to DDR in HCC, we further verified the role of WTAP in mediating CDDP sensitivity in HCC cells. We first performed immunofluorescence in WTAP-knockdown HCC cells and found that the formation of γH2AX foci was significantly increased following WTAP-knockdown and exposure to CDDP (Fig. 6A, B). Moreover, WTAP-knockdown inhibited the proliferation of HCC cells exposed to CDDP, DOX or ETOP (Fig. 6C–E). Coincidently, WTAP knockdown sensitized HCC cells to apoptosis induced by CDDP (Fig. 6F). Results of colony staining showed that the colony formation capacity of WTAP-knockdown cells exposed to CDDP was markedly reduced (Fig. 6G). We further investigated the role of WTAP in CDDP resistance in vivo. The subcutaneous xenograft tumor model in nude mice showed a significant decrease in tumor size and weight, indicating that downregulation of WTAP inhibited the growth of CDDP-exposed HCC cells (Fig. 6H–J). These findings reveal that WTAP knockdown sensitizes HCC cells to chemotherapeutic drugs that induce DSB.

## Discussion

Ultraviolet (UV) radiation can cause rapid and short-term m^6^A modification of the RNAs corresponding to the DNA damage site, which is regulated by METTL3 and FTO, indicating that m^6^A is crucial for the repair of UV-induced pyrimidine dimers [25]. Although WTAP is not directly recruited to DNA damage sites to participate in the DNA damage repair process, it may indirectly contribute to DDR by regulating other DNA damage response proteins. Additionally, the relationship between m6A modification and other types of DNA damage has not been explored. In addition, it is documented that knockout of FTO can alter the expression of certain genes in the DDR pathway in osteoblasts [26], and that the upregulation of FTO can modulate the expression of β-catenin by reducing the m^6^A level in mRNA transcripts, thereby enhancing tumor cell resistance to chemoradiation [27]. Silencing of METTL3 can weaken DDR and strengthen the sensitivity to gamma radiation [28, 29]. Collectively, these studies suggest that m^6^A modification is related to DDR. However, the specific mechanisms of how m^6^A-related proteins involved, except for METTL3 and FTO, remain to be clarified.

**Fig. 7:**
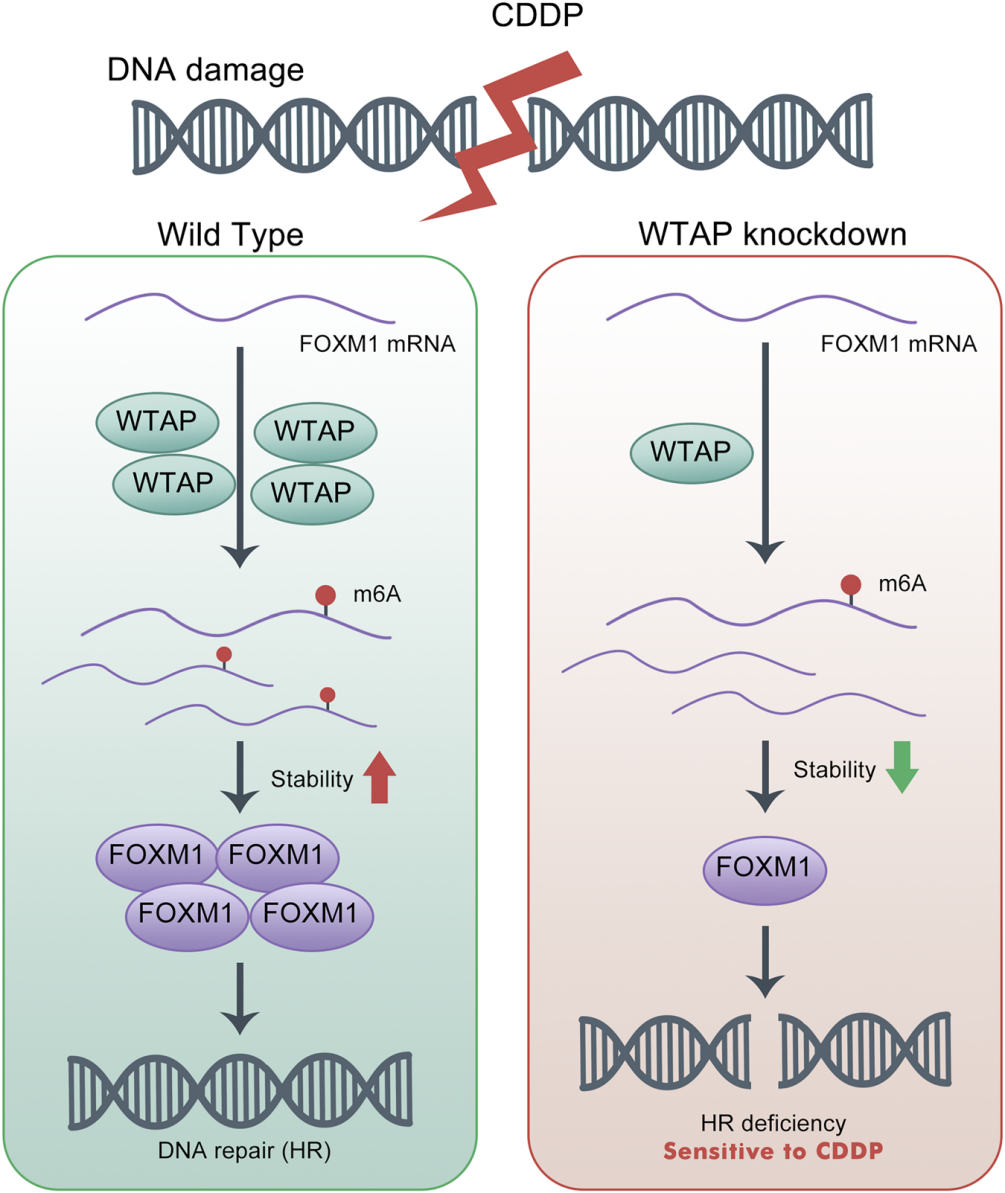
Molecular mechanism of WTAP involved in DDR. The m6A methylation level of FOXM1 mRNA increases in the process of CDDP-induced DNA damage in HCC due to the high expression of WTAP, thereby advancing FOXM1 mRNA stability and leading to the subsequent elevation of FOXM1 protein expression, and eventually promoting HR efficiency.

In this study, we first demonstrated that WTAP was significantly upregulated in HCC cells following exposure to three well-known DNA damaging agents, CDDP, DOX and ETOP. Further analysis demonstrated that following CDDP-induced DNA damage, the m^6^A methylation level of a series of WTAP-dependent genes was significantly altered. These genes were significantly enriched in the DNA double-strand break repair pathway, suggesting that m^6^A modification, especially WTAP, might play a synergistic role in DDR. We further found that the m^6^A level of FOXM1, a transcription factor closely related to DDR [23, 24], was regulated by WTAP and CDDP. In the case of the induction of DNA damage and WTAP-knockdown, the m^6^A modification level of FOXM1 was notable decreased, which was due to the impaired stability of its mRNA. In general, our results indicate that the m^6^A methylation level of FOXM1 mRNA increases following CDDP-induced DNA damage in HCC cells due to the high expression of WTAP, thereby increasing FOXM1 mRNA stability and leading to the subsequent elevation of FOXM1 protein expression, and eventually promoting HR efficiency (Fig. 7).

FOXM1 nascent transcripts were reported to be substrates of the m^6^A demethylase ALKBH5 [18]. ALKBH5 could enhance the expression of FOXM1 by demethylating FOXM1 nascent transcripts [18]. However, the SUMOylation of ALKBH5 was demonstrated to be promoted during reactive oxygen species (ROS)-induced DNA damage, which led to inhibition of ALKBH5 m^6^A demethylase activity by blocking substrate accessibility [30]. The m^6^A-Modified RNA was observed to localize to DNA damage sites and the global mRNA m6A modification was markedly upregulated following induction of DNA damage [19, 25]. Our findings demonstrated increased WTAP expression as well as FOXM1 m^6^A methylation levels following induction of DNA damage, leading to enhanced mRNA stability and protein expression of FOXM1. Therefore, the discrepancy of ALKBH5 and WTAP in regulating FOXM1 might be caused by the inhibition of ALKBH5 m^6^A demethylase activity and promotion of WTAP-medicated FOXM1 m^6^A methylation following induction of DNA damage.

The proliferation-specific oncogenic transcription factor FOXM1 is critical in multiple processes of DNA damage response, regulating the transcription of DNA damage sensing, mediating, signaling and repair genes (BRCA2, EXO1, RAD51, BRIP1, XRCC1/2, NBS1) [31]. FOXM1 is an upstream transcriptional stimulator of BRCA2 and the recombinase RAD51 that can be recruited onto the DNA damage sites, inducing strand invasion during HR [21]. NBS1 is a direct transcriptional target of FOXM1, and FOXM1 has been reported to regulate the expression of NBS1 through a forkhead response element site within its promoter and thus MRN (MRE11/RAD50/NBS1) complex formation to promote ATM activation and phosphorylation, modulating HR DNA damage repair [22]. In the current study, we observed that WTAP significantly reduced the protein expression of RAD51 and NBS1, but had no obvious effect on ATM expression. Moreover, results from our current work and previous studies have demonstrated that FOXM1 promoted the expression of RAD51 and NBS1 [21, 22]. Overall, these findings indicated that RAD51 and NBS1 might be involved in the WTAP-FOXM1-mediated DDR. The detailed mechanisms underlying WTAP-FOXM1-mediated DDR and whether FOXM1 functions in other pathways to impact DDR still require further investigation.

Our results showed that WTAP was markedly upregulated in HCC cells, and that it played an oncogenic role in HCC. Additionally, we revealed that the expression of WTAP further increased during the induction of DSB by CDDP, DOX and ETOP. As documented in a previous article describing the involvement of METTL3 in HR, the induction of DNA damage results in the activation of ATM, which subsequently promotes the phosphorylation of METTL3. Phosphorylated METTL3 is assigned to the side of DNA damage and modifies the nascent RNAs to promote HR. In-depth studies are required to explore the detailed molecular mechanisms of the increase in WTAP expression following the induction of DNA damage.

In conclusion, the present study was the first to delineate the involvement of WTAP-m^6^A in the DDR by promoting m^6^A modification of FOXM1 mRNA and subsequently enhancing stabilization of the transcript. In addition, WTAP deficiency sensitized HCC cells to CDDP both in vitro and in vivo, indicating that WTAP may be a novel therapeutic target for patients with HCC.

## Materials and methods

### Cells

HEK293T and HCC cell lines (BEL-7402 and SMMC-7721) were purchased from the Cell Bank of the Chinese Academy of Sciences (Shanghai, China) and grown in Dulbecco’s modified Eagle’ medium (DMEM, Gibco) containing 10% fetal bovine serum (Gibco), 100 U/mL penicillin streptomycin (Gibco). The culture dishes were placed in an incubator with a constant temperature of 37 °C and a mixed gas of 5% CO_2_ and 95% O_2_. BEL-7402 and SMMC-7721 used in our study were identified by STR analysis (Genechem co., Ltd. Shanghai, China). The cell lines STR profile reports have been submitted as supplementary materials in our paper.

### Oligonucleotides and vectors

Small interfering RNAs (siRNAs) against WTAP and the negative control were chemically synthesized by GenePharma (Suzhou, China). Lentivirus-based short hairpin RNA (shRNA) vector against WTAP, the overexpression plasmids of FOXM1 and the respective control plasmids purchased from Ke Lei Biological Technology (Shanghai, China) were used to construct stable interfering and overexpressing cells. All the sequences were listed in detail in Table S2. Transfection using Lipofectine2000 (Invitrogen, USA) and lentivirus infection were performed as described previously [32].

### Quantitative real-time PCR (RT-qPCR)

Total RNA was isolated using TRIzol reagent (Invitrogen) following the manufacturer’s protocol. After quantification, 500−1000 ng of RNA was converted into cDNA using PrimeScript™ RT Regent Kit (TAKARA, Dalian, China). The mRNA expression levels were analyzed by quantitative PCR using TB Green® Premix Ex Taq™ II (Tli RNaseH Plus) (TAKARA). Sequences of primers used in RT-qPCR were listed in Table S3.

### Immunofluorescence assay

All specific manipulations were performed as previous studies [32].

### Detection of global m^6^A level

The global level of m^6^A RNA methylation in cells was detected using m^6^A RNA Methylation Quantification Kit (Abcam, # ab185912) according to the manufacturer’s instructions.

For dot blot assay used to evaluate the global m^6^A level, total RNA was extracted and diluted to 500 ng/μl with the RNA incubation buffer. Then, the RNAs were denatured at 95 °C for 5 min, followed by spotting onto Hybond N+ membranes (GE Healthcare, USA). The membranes were crosslinked by ultraviolet and blocked with 5% nonfat milk for 30 min, followed by incubating with anti-m6A (Synaptic Systems, #202003) at 4 °C overnight. After that, the membranes were incubated with HRP-conjugated horseradish peroxidase-conjugated anti-rabbit immunoglobulin G for 60 min at room temperature and dot blots were measured by an ECL imaging system (Bio-Rad, USA).

### Neutral comet assay

BEL-7402 and SMMC-7721 cells with knockdown of WTAP and the corresponding control were collected and mixed with 0.5% low-melting point agarose, followed by being added to the slides covered with 1% normal-melting agarose. After solidifying at 4 °C for 20 min, the slides were immersed in the iced neutral lysis buffer at 4 °C for 4 h. Then, the electrophoresis was performed with untwisted DNA in the pre-cooled neutral electrophoresis buffer. Finally, the slides were stained with SYBR Green I and photographed by the inverted fluorescence microscope (Carl Zeiss). DNA damage was visualized as the percentage of tail DNA and tail moment, analyzed using CASP software with 50 cells analyzed per group.

### RNA immunoprecipitation (RIP)-qPCR

RNA immunoprecipitation was performed using EZ-Magna RIP™ RNA-Binding Protein Immunoprecipitation Kit (#17-701, Merk Millipore, MA, USA) according to the manufacturer’s instructions.

### RNA stability assay

To investigate the lifespan of FOXM1, the actinomycin (5 μg/ml, Sigma) was used to stop transcription of cells. Cells were then collected 0, 2, 4, 8, and 10 h after treatment. The total RNA was isolated and the remaining FOXM1 was detected using RT-qPCR.

### Colony formation assay

All specific manipulations were performed as previous studies [32].

### Cell apoptosis assay

According to previous manipulations [32], cell apoptosis assay was performed using apoptosis detection kits (556547, BD Biosciences, USA) and (A214-01, Vazyme, China). The cells were then analyzed with the BD LSRFortessa Flow Cytometer System (BD Biosciences) and FlowJo software.

### Xenograft tumor model

Animal studies were approved by the Animal Research Ethics Committee of Shanghai Tenth People’s Hospital (SHDSYY-2022-6118). The male BALB/c nude mice (5 week-old) were purchased from the Beijing Vital River Laboratory Animal Technology Co. Ltd. (Beijing, China). Mice were randomly divided into two groups based on body weight. BEL-7402 cells with stable knockdown of WTAP (shWTAP) and corresponding control cells (5 × 10^6^ cells) were subcutaneously injected into the mice. After inoculation of 7 days, the formation of transplanted tumor was observed, and the tumor length and width were measured with a vernier caliper every week and calculated as follows: volume (mm^3^) = (length × width^2^) × 0.5. The mice were anesthetized and then sacrificed after 4–5 weeks. Then tumors were isolated to check their weight. For drug sensitivity assay, most of the steps were the same as mentioned above. When the tumors grew to a measurable size, the mice (5 mice per group) were randomly intraperitoneally injected with saline (100 μl) or 5 mg/kg CDDP (dissolved in 100 μl saline). The injections were administered 3 times weekly for 2–3 weeks. For animal studies, blinding was not performed during the experiment.

### Western blotting assay

Western blotting assay was performed as previously described procedures [32]. The specific primary antibodies applied in this study were as follows: anti-WTAP (Abcam, # ab195380, # ab245628), anti-METTL3 (Abcam, # ab195352), anti-METTL14 (Abcam, # ab220030), anti-FTO (Abcam, # ab92821), anti-RBM15 (Abcam, # ab70549), anti-γH2AX (Abcam, # ab26350), anti-FOXM1 (Abcam, # ab207298), anti-NBS1 (Abcam, # ab32074), anti-RAD51(Abcam, # ab133534) and anti-GAPDH (CST, # 2118S) as internal reference.

### Statistical analysis

GraphPad Prism 7.0 and SPSS ver.20.0 software were used for data analysis and related diagraming. The data of three groups of independent experiments were expressed by mean ± STD. Student’s *t*-test and One-way ANOVA were used to compare the difference between two groups and multiple groups respectively. Statistical analysis of the Comet assays and gH2AX foci analyses was performed using the Mann-Whitney test. Statistical significance was marked as follows: *p < 0.05, **p < 0.01, ***p < 0.001.

## Supplementary information

Supplementary information

Figure S1. The overall m6A level and m6A methyltransferase mRNA expression after induction of DNA damage in HCC cells

Figure S2. The overall m6A level in HCC cells with the knockdown of WTAP

Figure S3

Figure S4. WTAP is highly expressed in various tumors and is related to the prognosis of patients with HCC

Figure S5 The oncogenic role of WTAP in HCC

supplemental material table S1

supplemental material table S2

supplemental material table S3

supplemental material WB original data

## Data Availability

The data that support the findings of this study are available from the corresponding author upon reasonable request.
