# Supplementary material for: WTAP participates in the DNA damage response via an m6A-FOXM1-dependent manner in hepatocellular carcinoma
Source: Cell Death Discov. 2025 Aug 22;11:397. doi: 10.1038/s41420-025-02639-x (PMC12373989; doi:10.1038/s41420-025-02639-x)
Supplement: Supplementary file 1 — Supplementary information [file 41420_2025_2639_MOESM1_ESM.doc]

**Supplementary information**

**Table S1. The specific differentially methylated genes in different groups.**

**Table S2. Target sequences of siRNAs used in this work.**

**Table S3. RT-PCR primers used in this study.**

**Figure S1. The overall m^6^A level and m^6^A methyltransferase mRNA expression after induction of DNA damage in HCC cells.**

**A-B** Dot blot assay used to evaluate the overall m6A level in HCC cells. **B**, treated with three DNA damage inducing drugs (10 μg/ml CDDP for 12 h, 5 μM DOX for 12 h and 50 μM ETOP for 16 h). The mRNA expression of METTL3 and METTL14 in BEL-7402 and SMMC-7721 HCC cells treated with CDDP (10 μg/ml for 12 h) **C, E** or Etop (50 μM for 16 h) **D, F**.

**Figure S2. The overall m6A level in HCC cells with the knockdown of WTAP.**

**A** Western blot analysis of WTAP protein expression in a panel of HCC cell lines and the normal liver cell line. **B** Dot blot assay was used to evaluate the overall m6A level in HCC cells with stable knockdown of WTAP (shWTAP) or the corresponding control (shCON).

**Figure S3. FOXM1 is involved in DDR.**

**A** Immunofluorescence analysis of co-localization of WTAP and γH2AX in BEL-7402 cells. **B** The protein expression of NBS1, RAD51 and γH2AX in FOXM1-deficient cells with CDDP treatment. **C** Immunofluorescence analysis of the formation of γH2AX foci in FOXM1-knockdown cells with CDDP treatment (n=50, Mann-Whitney test).

**Figure S4. WTAP is highly expressed in various tumors and is related to the prognosis of patients with HCC.**

**A** Boxplots represented WTAP mRNA expression level in various and paired non-tumor tissues. **B** Immunohistochemical analysis of WTAP in normal liver tissue and HCC tissue provided by the Human Protein Atlas project. Scale bar, 50 μm. **C** Kaplan–Meier survival curves of overall survival in 370 HCC patients based on WTAP expression level. **D** Relative expression of WTAP mRNA transcript in normal and grade 1, 2, 3 and 4 HCC patients. **E** Relative expression of WTAP mRNA transcript in normal and stage N0, N1 HCC patients.

**Figure S5 The oncogenic role of WTAP in HCC.**

**A-B** BEL-7402 and SMMC-7721 cells were infected with lentivirus-based shRNA against WTAP to establish stable WTAP-knockdown cells. The silencing efficiency was verified using western blot and RT-qPCR. **C-D** CCK8 assays, **E** colony staining, and **F** flow cytometry utilized to evaluate the role of WTAP in cell proliferation, colony formation capacity and apoptosis respectively. **G** Subcutaneous tumor xenograft model of BEL-7402 cells with or without WTAP knockdown. **H-I** Tumor volume and weight were measured at indicated day points after cells were transplanted into mice. All the results were obtained from at least three independent experiments. ***p*<0.01, ****p*<0.001 compared with shCON, or siCON.
