# Supplementary figures and images for: WTAP participates in the DNA damage response via an m6A-FOXM1-dependent manner in hepatocellular carcinoma
Source: Cell Death Discov. 2025 Aug 22;11:397. doi: 10.1038/s41420-025-02639-x (PMC12373989; doi:10.1038/s41420-025-02639-x)

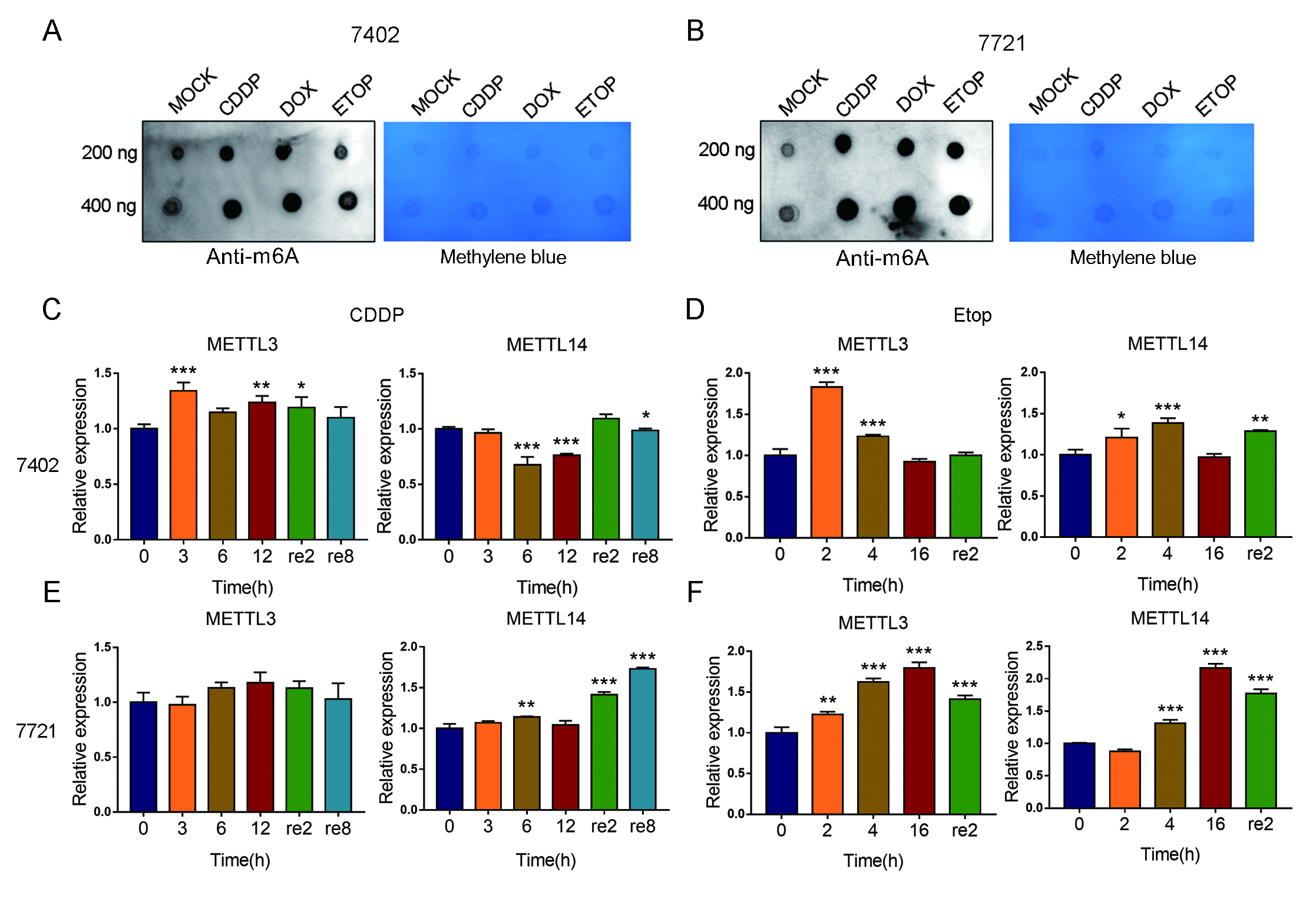

Supplement: Supplementary file 2 — Figure S1. The overall m6A level and m6A methyltransferase mRNA expression after induction of DNA damage in HCC cells [file 41420_2025_2639_MOESM2_ESM.tif]

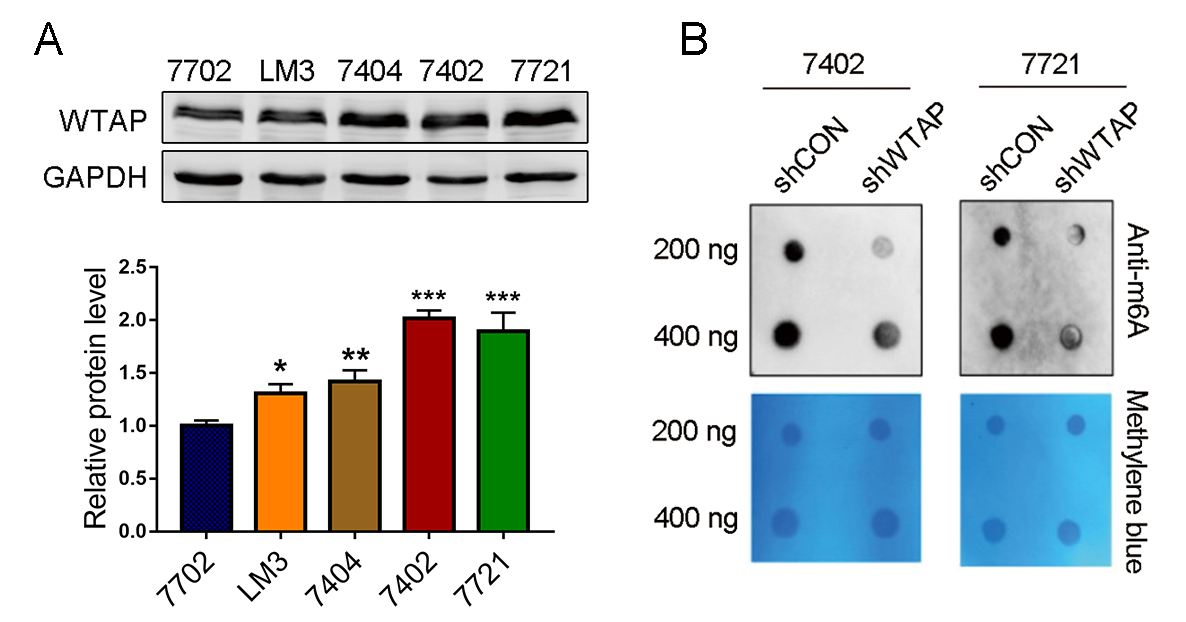

Supplement: Supplementary file 3 — Figure S2. The overall m6A level in HCC cells with the knockdown of WTAP [file 41420_2025_2639_MOESM3_ESM.tif]

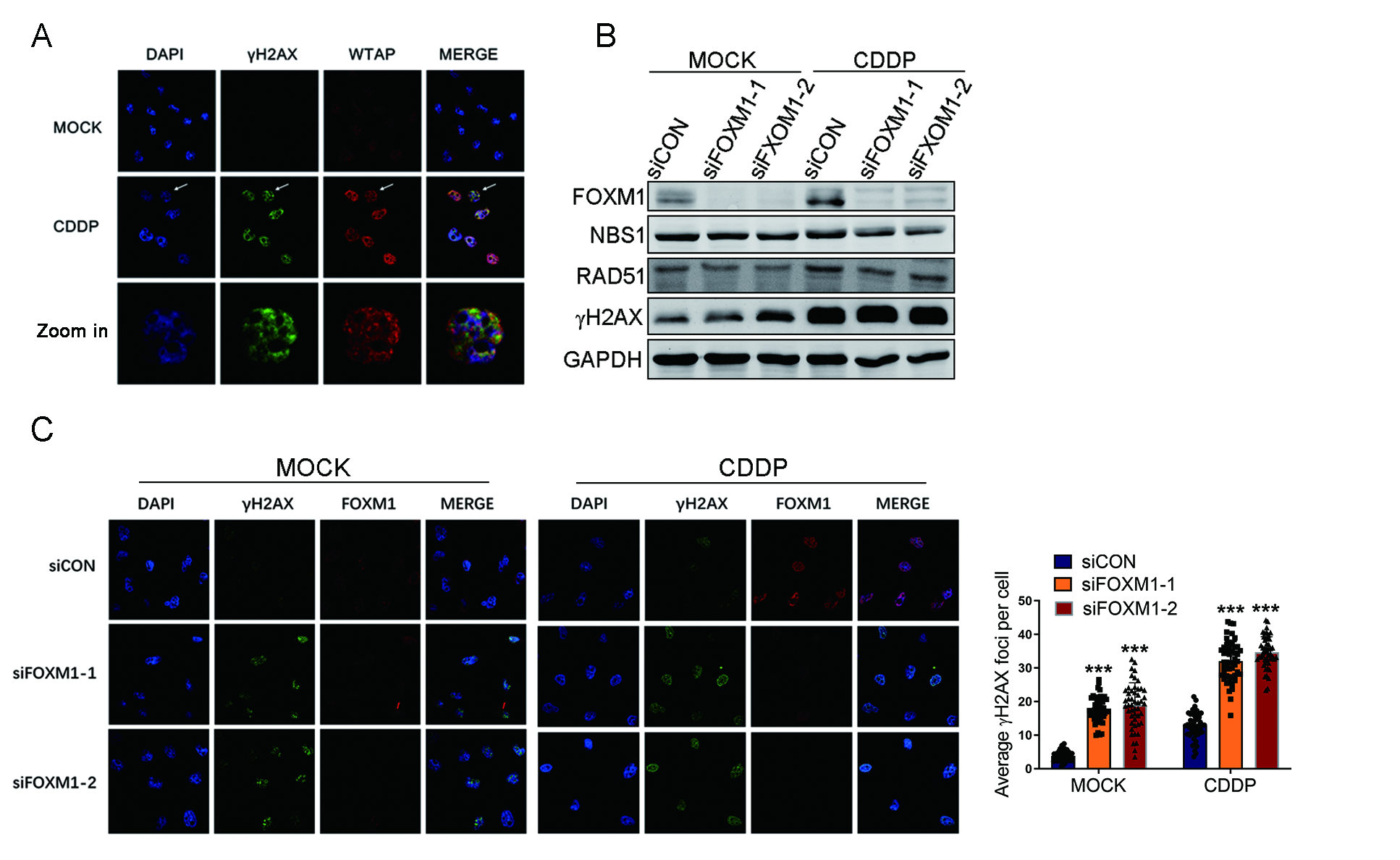

Supplement: Supplementary file 4 — Figure S3 [file 41420_2025_2639_MOESM4_ESM.tif]

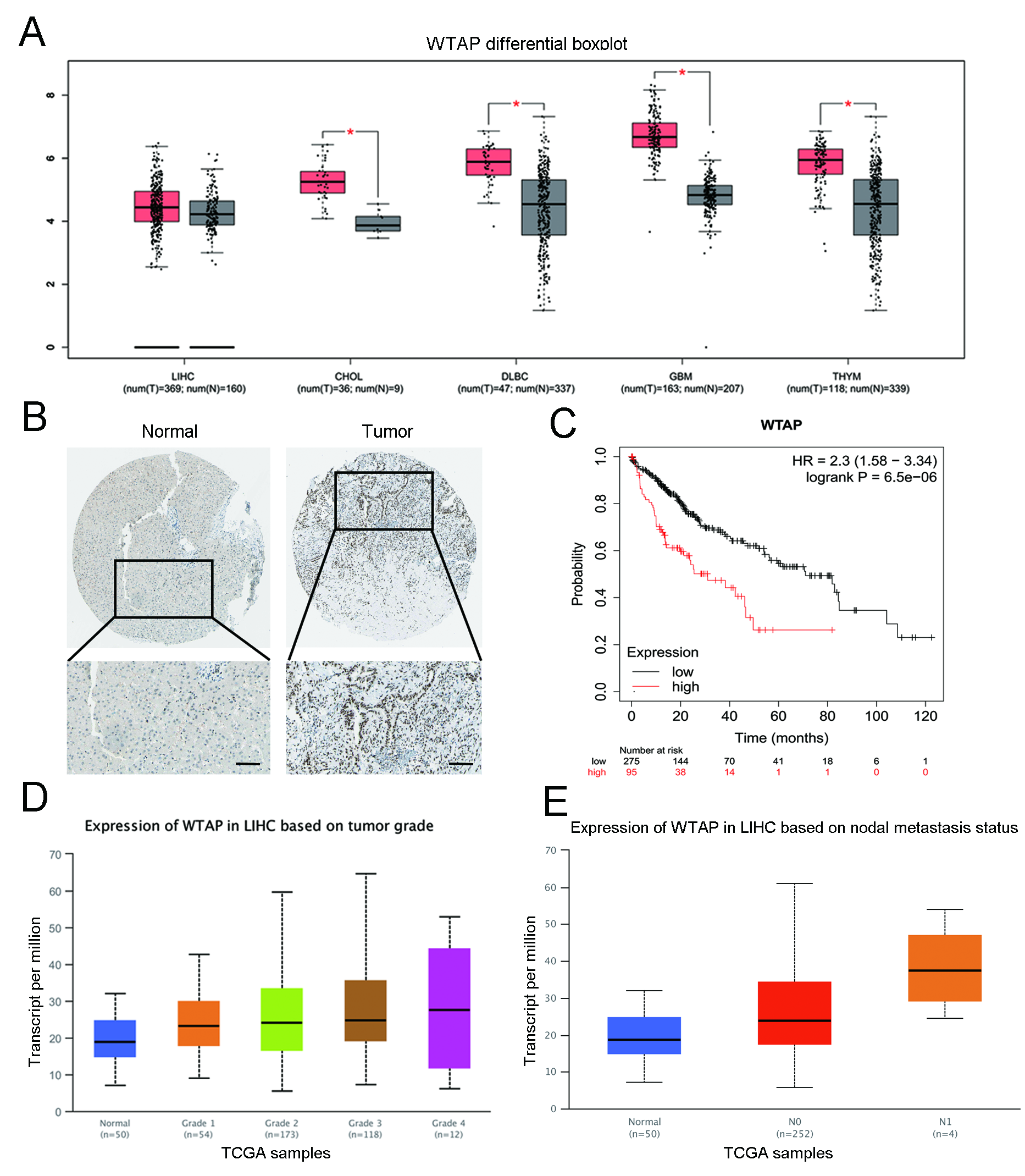

Supplement: Supplementary file 5 — Figure S4. WTAP is highly expressed in various tumors and is related to the prognosis of patients with HCC [file 41420_2025_2639_MOESM5_ESM.tif]

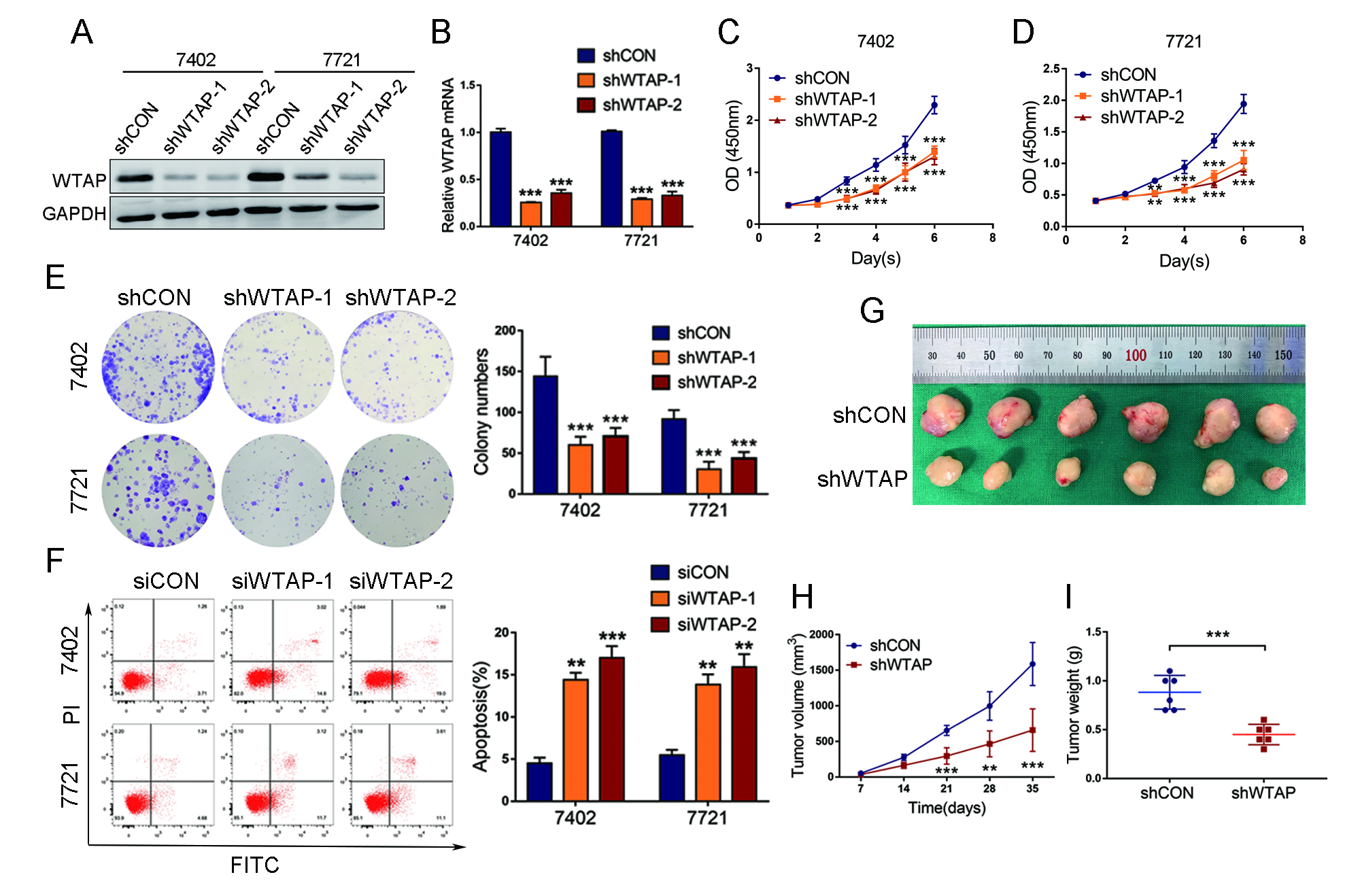

Supplement: Supplementary file 6 — Figure S5 The oncogenic role of WTAP in HCC [file 41420_2025_2639_MOESM6_ESM.tif]
