# Supplementary material for: WTAP participates in the DNA damage response via an m6A-FOXM1-dependent manner in hepatocellular carcinoma
Source: Cell Death Discov. 2025 Aug 22;11:397. doi: 10.1038/s41420-025-02639-x (PMC12373989; doi:10.1038/s41420-025-02639-x)
Supplement: Supplementary file 8 — supplemental material table S2 [file 41420_2025_2639_MOESM8_ESM.docx]

| **Gene symbol** | **Targeted sequence** |
| --- | --- |
| siWTAP-1 | GCAAGAGUGUACUACUCAA |
| siWTAP-2 | GCCCAACUGAGAUCAACAA |
| shWTAP | GCCCAACUGAGAUCAACAA |
| siFOXM1-1 | CTCTTCTCCCTCAGATATA |
| siFOXM1-2 | GGACCACTTTCCCTACTTT |

**Table S2. Target sequences of siRNAs used in this work.**
