# Supplementary material for: WTAP participates in the DNA damage response via an m6A-FOXM1-dependent manner in hepatocellular carcinoma
Source: Cell Death Discov. 2025 Aug 22;11:397. doi: 10.1038/s41420-025-02639-x (PMC12373989; doi:10.1038/s41420-025-02639-x)
Supplement: Supplementary file 9 — supplemental material table S3 [file 41420_2025_2639_MOESM9_ESM.docx]

**Table S3. RT-PCR primers used in this study.**

| Gene symbol | Forward primer | Reverse primer |
| --- | --- | --- |
| GAPDH | TGCACCACCAACTGCTTAGC | GGCATGGACTGTGGTCATGAG |
| WTAP | ACTGGCCTAAGAGAGTCTGAAG | GTTGCTAGTCGCATTACAAGGA |
| METTL3 | GGAATCACCTCCGACACTC | AAGCTGCACTTCAGACGAAT |
| METTL14 | AGTGCCGACAGCATTGGTG | GGAGCAGAGGTATCATAGGAAGC |
| FTO | AACACCAGGCTCTTTACGGTC | TGTCCGTTGTAGGATGAACCC |
| ALKBH5 | CGGCGAAGGCTACACTTACG | CCACCAGCTTTTGGATCACCA |
| FOXM1 | CGTCGGCCACTGATTCTCAAA | GGCAGGGGATCTCTTAGGTTC |
| MeRIP-FOXM1 | CCTGCAGTGAAGAACCCAAGA | ATTATCCAGAGACTGCCAGAAGG |
