# Supplementary material for: WTAP participates in the DNA damage response via an m6A-FOXM1-dependent manner in hepatocellular carcinoma
Source: Cell Death Discov. 2025 Aug 22;11:397. doi: 10.1038/s41420-025-02639-x (PMC12373989; doi:10.1038/s41420-025-02639-x)
Supplement: Supplementary file 10 — supplemental material WB original data [file 41420_2025_2639_MOESM10_ESM.pptx]

## Slide 1
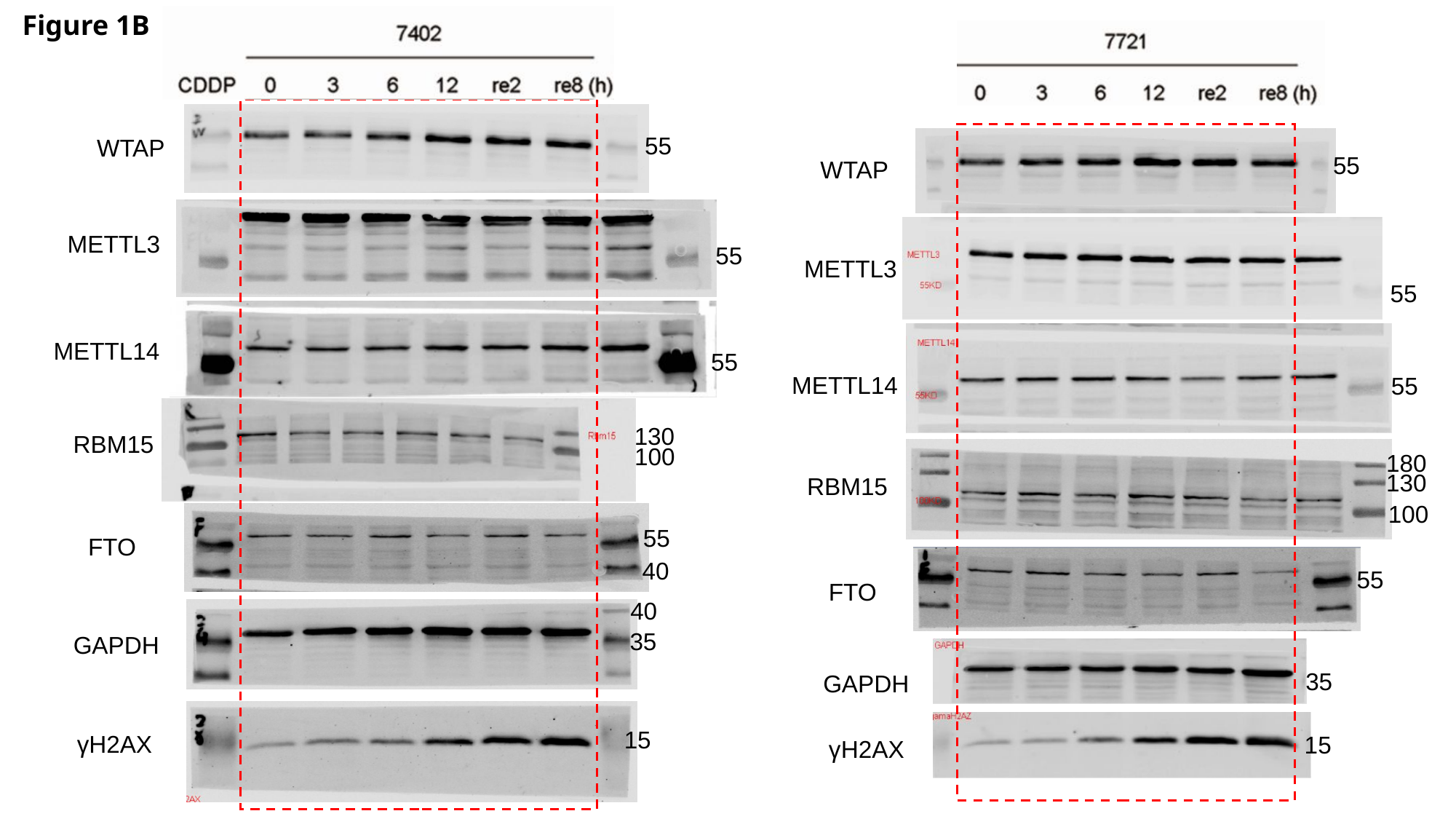

Figure 1B
55
WTAP
55
WTAP
METTL3
55
METTL3
55
METTL14
55
METTL14
55
130
RBM15
100
180
130
RBM15
100
55
FTO
40
55
FTO
40
35
GAPDH
35
GAPDH
15
γH2AX
15
γH2AX

## Slide 2
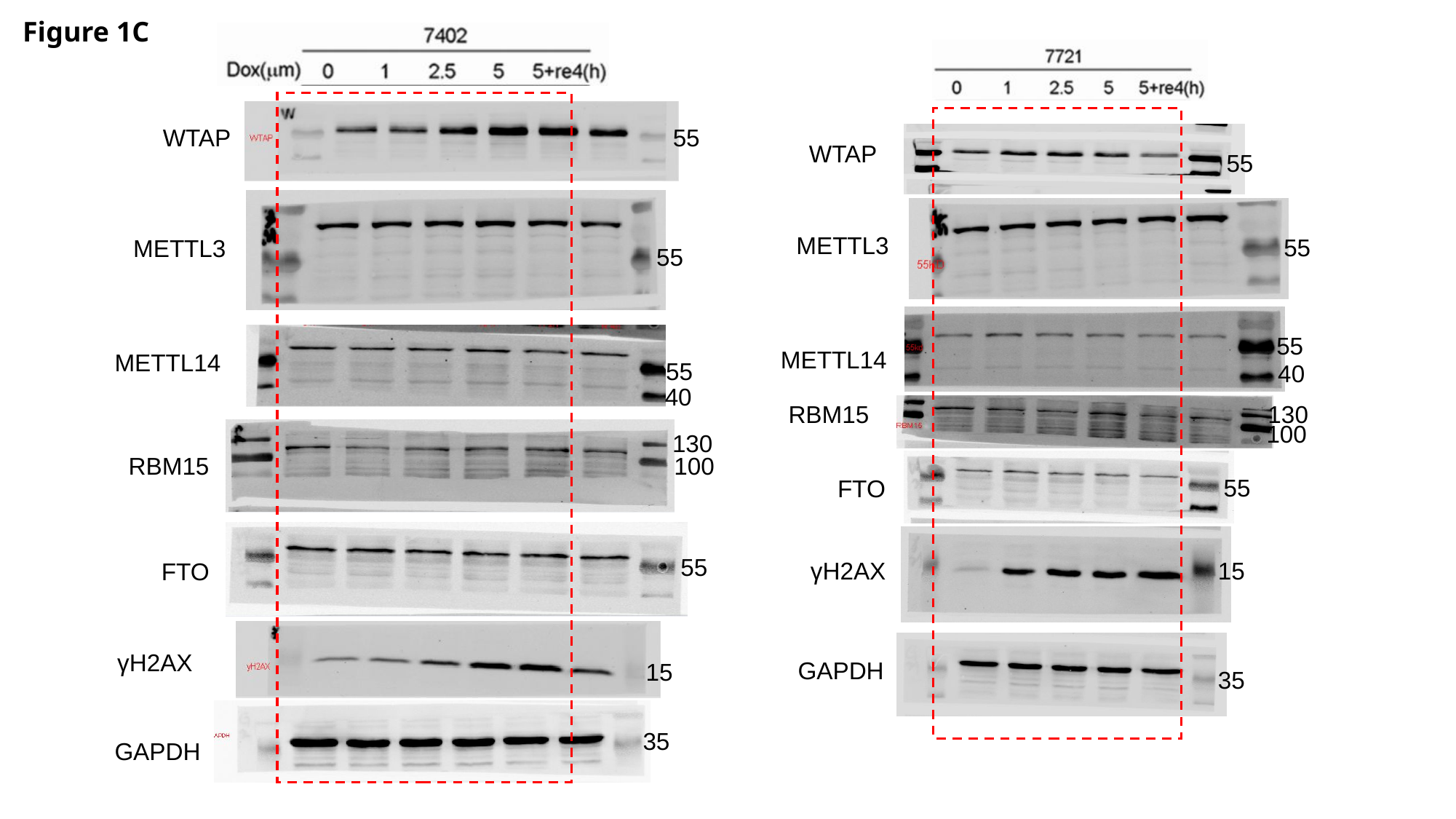

Figure 1C
WTAP
55
WTAP
55
METTL3
55
METTL3
55
55
METTL14
METTL14
55
40
40
RBM15
130
100
130
RBM15
100
55
FTO
55
γH2AX
15
FTO
γH2AX
GAPDH
15
35
35
GAPDH

## Slide 3
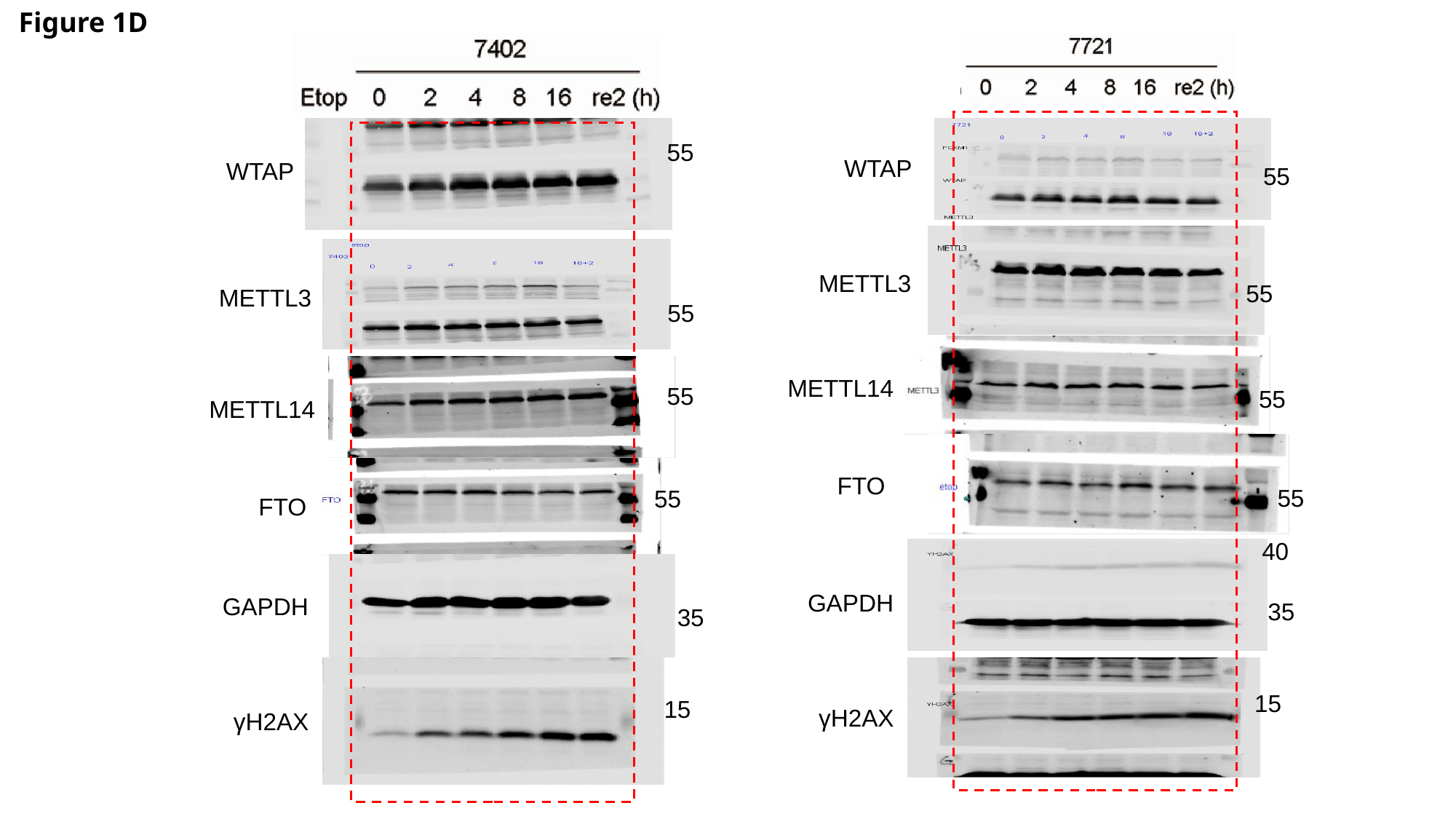

Figure 1D
55
WTAP
WTAP
55
METTL3
55
METTL3
55
METTL14
55
55
METTL14
FTO
55
55
FTO
40
GAPDH
GAPDH
35
35
15
15
γH2AX
γH2AX

## Slide 4
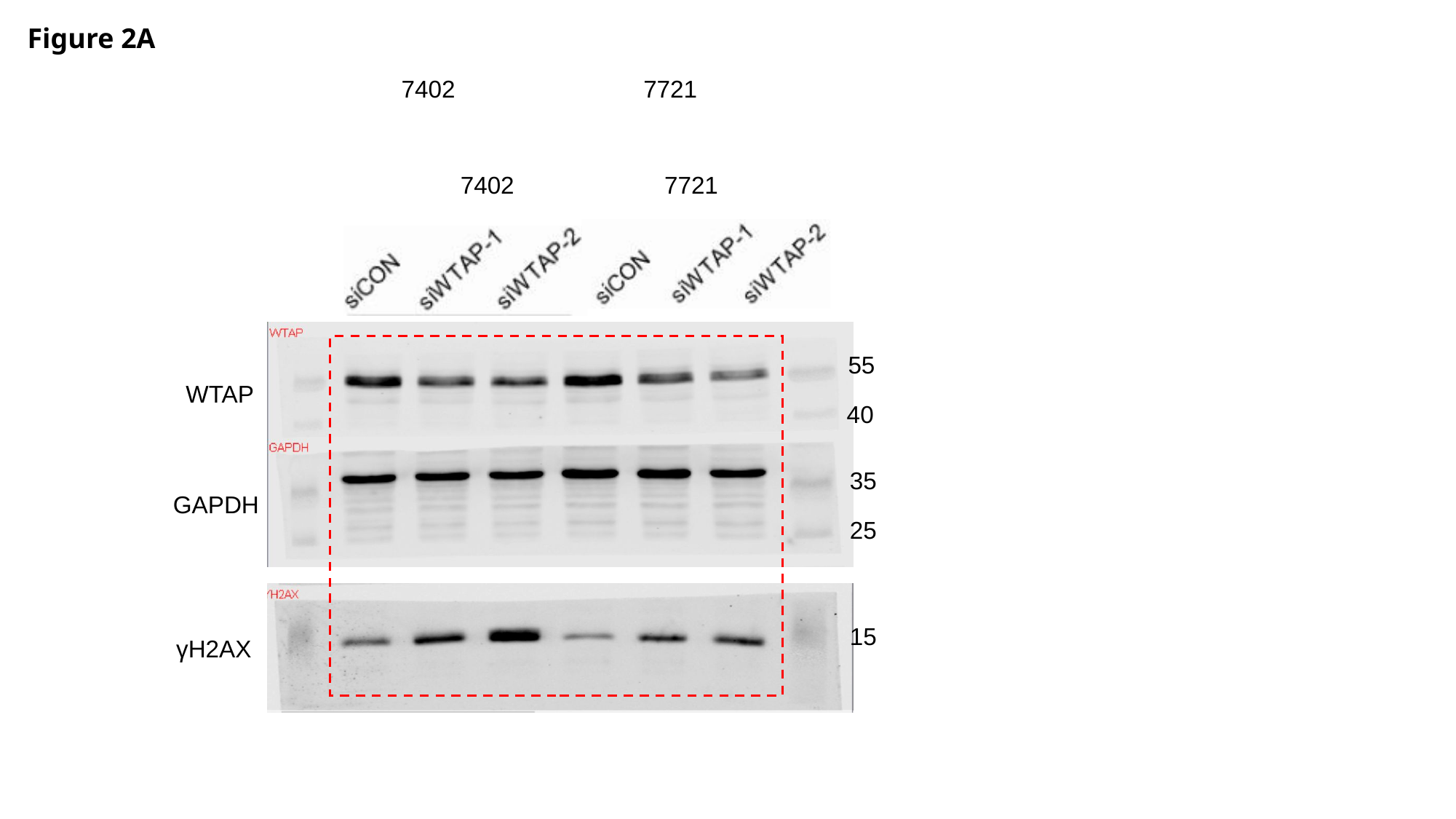

Figure 2A
7402
7721
7402
7721
55
WTAP
40
35
GAPDH
25
15
γH2AX

## Slide 5
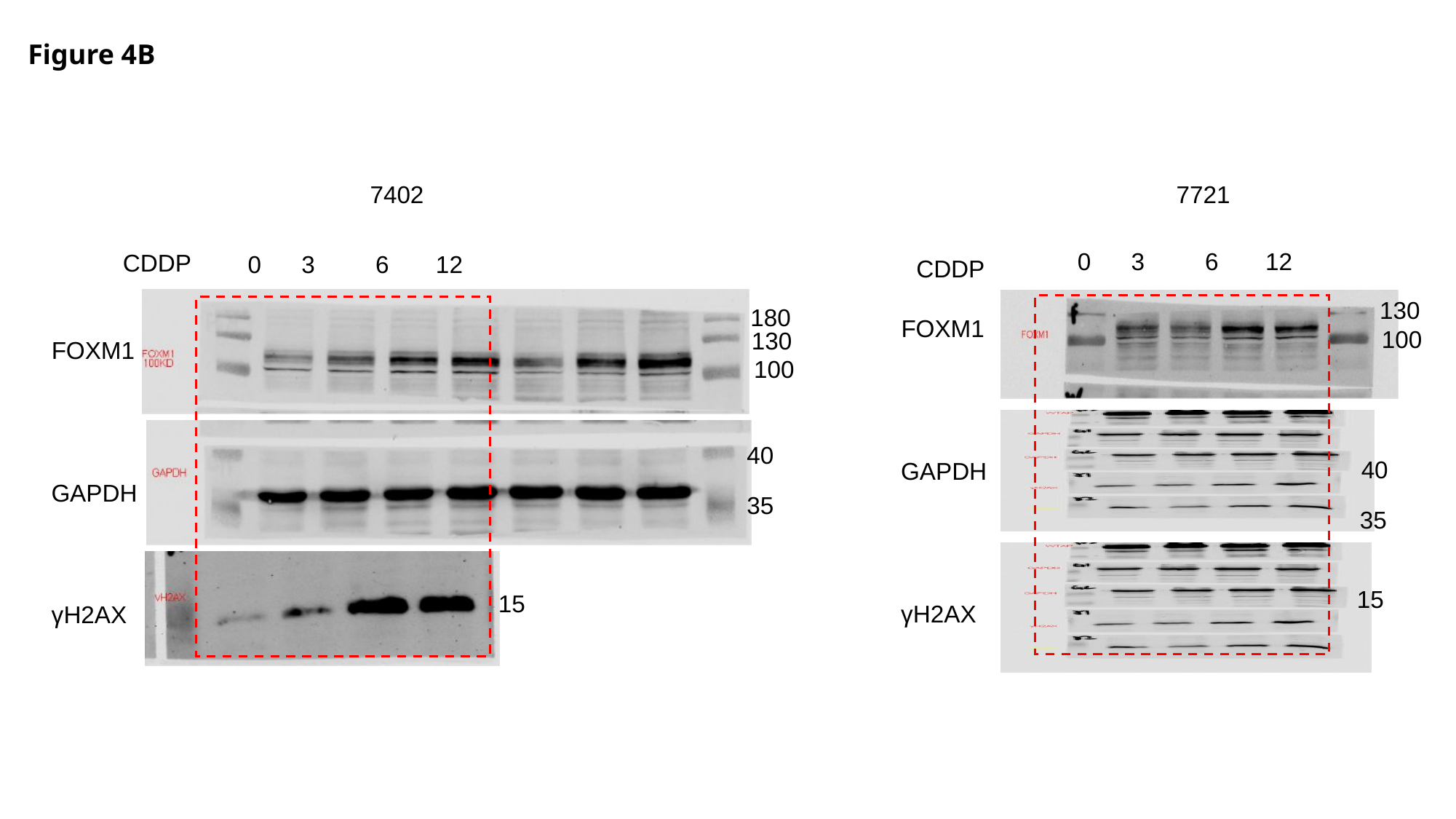

Figure 4B
7402
7721
0 3 6 12
CDDP
0 3 6 12
CDDP
130
180
FOXM1
100
130
FOXM1
100
40
40
GAPDH
GAPDH
35
35
15
15
γH2AX
γH2AX

## Slide 6
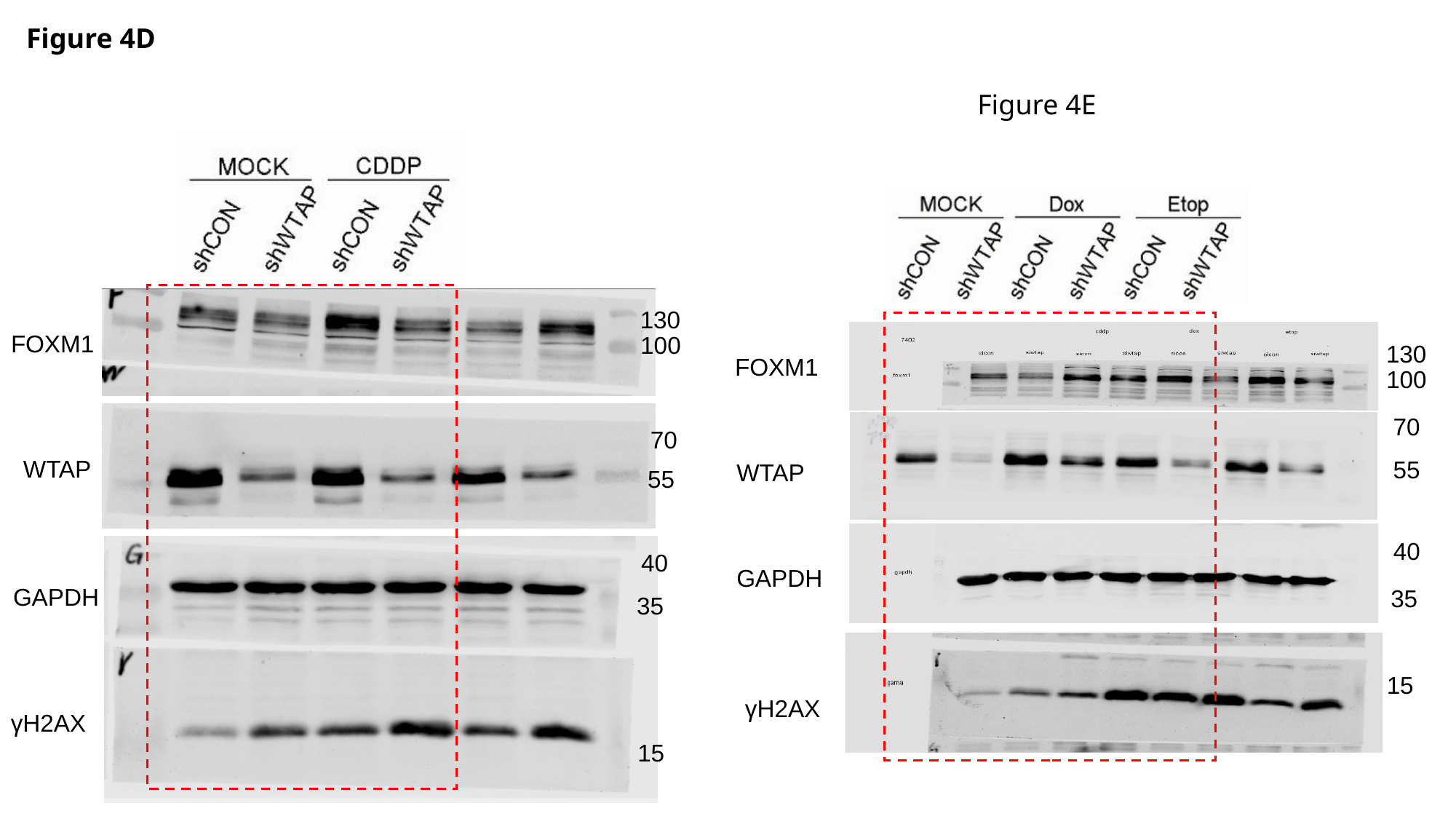

Figure 4D
Figure 4E
130
FOXM1
100
130
FOXM1
100
70
70
WTAP
55
WTAP
55
40
40
GAPDH
GAPDH
35
35
15
γH2AX
γH2AX
15

## Slide 7
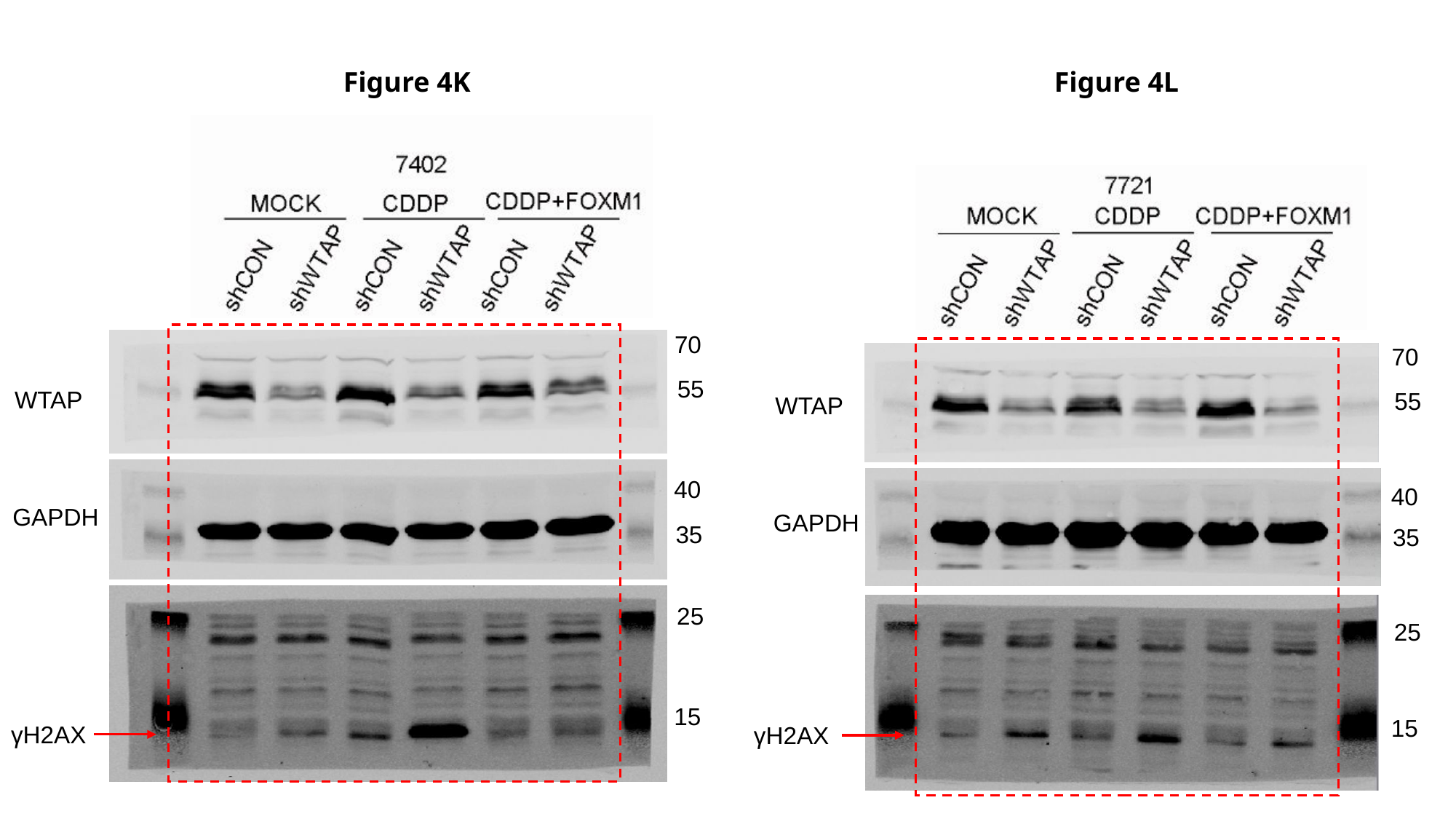

Figure 4K
Figure 4L
70
70
55
WTAP
55
WTAP
40
40
GAPDH
GAPDH
35
35
25
25
15
15
γH2AX
γH2AX
